# A Robust Synthesis of Fluorosurfactants with Tunable Functionalities via a Two‐Step Reaction

**DOI:** 10.1002/advs.202511811

**Published:** 2025-11-03

**Authors:** Jiyuan Yao, Shijian Huang, Shuting Xie, Zhenping Liu, Yueming Deng, Luca Carnevale, Mingliang Jin, Loes Irene Segerink, Da Wang, Lingling Shui, Sergii Pud

**Affiliations:** ^1^ International Joint Laboratory of Optofluidic Technology and System (LOTS) National Center for International Research on Green Optoelectronics Guangdong Provincial Key Laboratory of Nanophotonic Functional Materials and Devices, South China Academy of Advanced Optoelectronics & School of Optoelectronic Science and Engineering South China Normal University Guangzhou 510006 P. R. China; ^2^ BIOS Lab‐on‐a‐chip group EEMCS Faculty MESA+ institute University of Twente Enschede 7500 AE The Netherlands; ^3^ South China Academy of Advanced Optoelectronics South China Normal University Guangzhou 510006 P. R. China

**Keywords:** droplet microfluidics, fluorosurfactant, tunable functionalities, two‐step Reaction

## Abstract

Fluorosurfactant‐stabilized microdroplets hold significant promise for a wide range of applications, owing to their biological and chemical inertness. However, conventional synthetic routes for fluorosurfactants typically require multiple reaction steps and stringent conditions, such as high temperatures and anaerobic environments. This complexity poses a significant limitation to the development of fluorosurfactant synthesis and its subsequent applications in droplet‐based systems. In this work, a robust two‐step synthesis of fluorosurfactants with tunable functionalities is presented. Microdroplets stabilized by these fluorosurfactants exhibit enhanced stability and biocompatibility. Notably, these fluorosurfactants facilitate the formation of nanodroplets that efficiently transport and concentrate fluorophores with high selectivity. Furthermore, it is demonstrated that colloidal self‐assemblies with distinct structures can be engineered by modulating interactions between the fluorosurfactants and colloidal particles. The synthetic approach provides a strategy for the rapid production of functional fluorosurfactants under mild conditions, enabling droplet‐based microfluidic techniques with applications in biology and material science.

## Graphical abstract

The fluorosurfactants are synthesized through a two‐step reaction, and demonstrated excellent performance in droplet microfluidics. In addition, they exhibited tunable functionalities at the droplet interface via hydrogen bonding and coordination interactions.

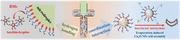

## 1 Introduction

Droplet microfluidics stands as a versatile technology for micrometer‐scale total analysis systems (µTAS) and advancing science across diverse research fields. It enables the generation and manipulation of isolated droplets with volumes ranging from picoliters to nanoliters on micro‐chip.^[^ ^1^, ^2^ ^]^ Due to their excellent gas permeability and compatibility with polydimethylsiloxane (PDMS) and poly(methyl methacrylate) (PMMA)‐based microfluidic chips, fluorinated oil is commonly applied as a continuous phase in generating water‐in‐fluorinated oil (W/O_F_) microdroplets with adjustable diameters and components. The generated microdroplets have broad applications, including the construction of spheroids and organoids,^[^ ^3^, ^4^, ^5^ ^]^ single cell analysis,^[^ ^6^, ^7^ ^]^ genetic engineering,^[^ ^8^ ^]^ and the structuring of self‐assemblies with tunable functionalities.^[^ ^9^, ^10^, ^11^ ^]^

Surfactants play a crucial role in both the emulsification and stabilization of emulsions in droplet microfluidics.^[^ ^12^, ^13^ ^]^ In contrast to conventional surfactants (e.g., Span 80, Abil EM 90, Abil EM 180), fluorosurfactants, consisting of hydrophobic and oleophobic “fluorinated tail” and hydrophilic “head” groups, exhibit superior compatibility with fluorinated oil. This enhances their ability to prevent coalescence in water‐in‐fluorinated oil (W/O_F_) or organic solvent‐in‐fluorinated oil (S_org_/O_F_) microdroplets.^[^ ^9^, ^14^, ^15^ ^]^ Over the past decades, a variety of fluorosurfactants with different hydrophilic groups have been developed.^[^ ^16^, ^17^, ^18^, ^19^, ^20^ ^]^ A common strategy for synthesizing fluorosurfactants involves leveraging electrostatic interactions between polyetherdiamine additives in the aqueous phase and carboxylated perfluorocarbon surfactants in the fluorinated oil phase.^[^ ^21^ ^]^ However, this method often compromised the stability of fluorosurfactants. For example, polycationic biomarkers containing amine groups can be adsorbed at the droplet interface through weak electrostatic interactions, interfering with the formation of stable fluorosurfactants and affecting their performance in bioimaging and screening applications.^[^ ^16^, ^22^ ^]^ To address these limitations, covalently linking hydrophilic segments with fluorinated tails has emerged as an effective approach, leading to commercially available products such as PFPE(H)_2_‐PEG_600_ (RAN Biotechnologies) and Pico‐Surf (Dolomite Microfluidics, Royston, UK).^[^ ^20^ ^]^ However, the complex synthetic procedures — such as amide linkage,^[^ ^16^ ^]^ click chemistry,^[^ ^23^, ^24^ ^]^ and living radical polymerization,^[^ ^20^ ^]^ — along with the use of moisture‐sensitive reagents (e.g., thionyl chloride and perfluoropolyether‐based (PFPE) fragmentation chain transfer (RAFT) reagent), constrain their application in droplet microfluidics, as summarized in Table S1 (Supporting Information). Furthermore, the synthesis of customized fluorosurfactants with tunable functionalities, such as stimulus response,^[^ ^25^, ^26^ ^]^ or interfacial interactions,^[^ ^27^, ^28^ ^]^ remains largely unexplored. Therefore, there is a pressing need for a simpler, more efficient synthetic route for functional fluorosurfactants to advance droplet‐templating microfluidic techniques.

In this work, we introduce a two‐step synthetic strategy to prepare fluorosurfactants with tunable functionalities. Our approach begins with the synthesis of a customized fluorosurfactant PFPE(H)‐COOCOOC_2_H_5_, via a mixed anhydride reaction, followed by the amidation‐based synthesis of commercially available fluorosurfactants (PFPE(H)_2_‐ED_900_ and PFPE(H)_2_‐PEG_600_) through amidation reaction. This method offers several advantages over previous protocols, including reduced reaction time and milder reaction conditions. We demonstrate that these fluorosurfactants enhance microdroplet stability across a broad temperature range and exhibit excellent biocompatibility, facilitating applications such as yeast culturing and HepG2 cell aggregation within microdroplet. Moreover, we demonstrate that these fluorosurfactants enable tunable functionalities: 1) nanodroplets, formed via spontaneous emulsification, selectively transport and enrich fluorophores, and 2) two distinct superstructures were obtained through the self‐assembly of nanoparticles (NPs) from drying droplets, driven by interactions between the fluorosurfactants and NPs. Our synthetic strategy expands the toolbox for developing functional fluorosurfactants and opens new avenues for the design of fluorinated oil‐based droplet microfluidics with tunable properties.

## 2 Results and Discussion

### 2.1 Two‐Step Reaction Synthesis of Fluorosurfactants

**Figure 1:**
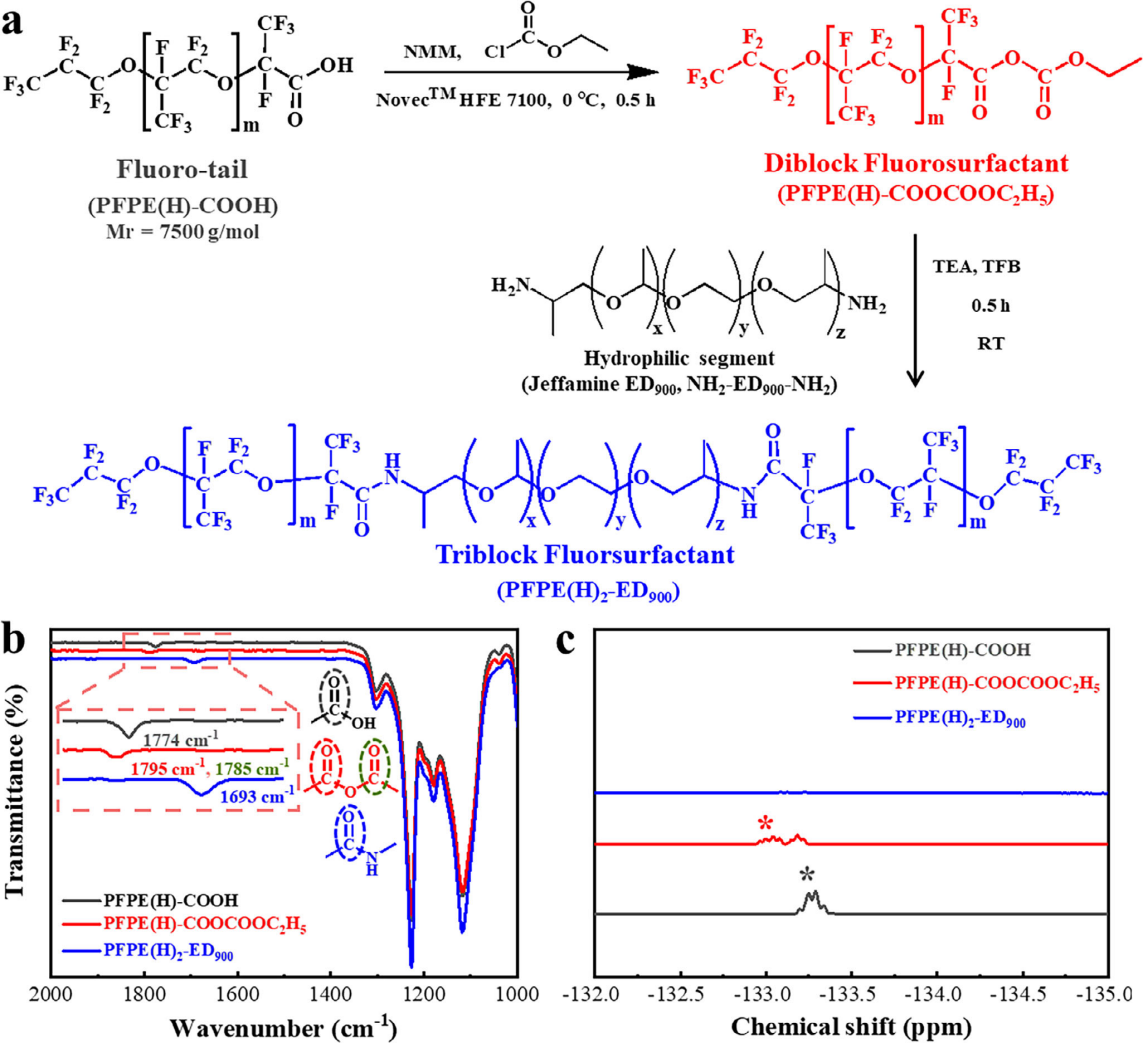
Synthesis and characterization of the fluorosurfactants. a) Schematic illustration of synthetic procedure for the fluorosurfactants in this study. b) FT‐IR spectra and c) ^19^F NMR spectra of PFPE(H)‐COOH, PFPE(H)‐COOCOOC_2_H_5_ and PFPE(H)_2_‐ED_900_ synthesized at RT for 0.5 h. Inset of panel b highlights the shift of the C═O stretch during the synthesis of the fluorosurfactants. Note that the asteroid in panel c denotes fluorine absorption vibration on the adjacent PFPE(H)‐COOH carboxyl carbon atom in these three fluorosurfactants.

We synthesized a diblock fluorosurfactant (PFPE(H)‐COOCOOC_2_H_5_) through a mixed anhydride reaction, coupling the fluorinated tail of carboxylated perfluoropolyether (PFPE‐COOH) and ethyl chloroformate at 0 °C for 0.5 h. Subsequently, triblock fluorosurfactant (PFPE(H)_2_‐ED_900_) was obtained via amidation reaction, coupling the PFPE(H)‐COOCOOC_2_H_5_ with hydrophilic segment Jeffamine ED_900_ (NH_2_‐ED_900_‐NH_2_) at room temperature (RT, 25 °C) for 0.5 h (**Figure** **1**a; see Supporting Information). Fourier‐transform infrared spectroscopy (FT‐IR) (Figure 1b) and ^19^F‐nuclear magnetic resonance (^19^F‐NMR) spectroscopy (Figure 1c; Figure S1, Supporting Information) were employed to characterize the chemical nature of the PFPE(H)‐COOH, PFPE(H)‐COOCOOC_2_H_5,_ and PFPE(H)_2_‐ED_900_, respectively. It is noted that carboxylic group (C═O) stretch at 1774 cm^−1^ in PFPE(H)‐COOH red‐shifted,^[^ ^18^ ^]^ and two new absorbance peaks at 1795 and 1785 cm^−1^ in PFPE(H)‐COOCOOC_2_H_5_ emerged, corresponding to the C═O stretch of the converted mixed anhydride. Upon coupling PFPE(H)‐COOCOOC_2_H_5_ with Jeffamine ED_900_, the C═O stretch at 1795 and 1785 cm^−1^ disappeared, and a new peak at 1693 cm^−1^ emerged, corresponding to the amide C═O stretch. The peak of ^19^F‐NMR signal at −133.25 ppm, appearing as a quartet, corresponds to the fluorine absorption vibration on the adjacent PFPE carboxyl carbon atoms.^[^ ^26^ ^]^ When ethyl chloroformate was covalently bound to the carboxyl group of PFPE(H)‐COOH, this peak shifted to −133.00 ppm. The peak disappeared upon coupling with the amino group of Jeffamine ED_900_ (NH_2_‐ED_900_‐NH_2_), confirming the formation of amide linkage in PFPE(H)_2_‐ED_900_. Both FT‐IR and ^19^F‐NMR spectroscopy results consistently confirm the successful synthesis of the fluorosurfactants.^[^ ^16^, ^26^ ^]^

The robustness of our proposed synthetic strategy at RT enables the incorporation of various hydrophilic segments with thermo‐sensitivity. We further demonstrated the successful synthesis of triblock fluorosurfactant (PFPE(H)_2_‐PEG_600_), using a PEG‐diamine hydrophilic segment, achieving a yield of ≈90% (Figure S2a–c, Supporting Information). Moreover, we scaled up the synthesis to 25.7 g of PFPE(H)_2_‐ED_900_ by proportionally increasing the reagents under identical reaction conditions (0.5 h for carboxyl activation at 0 °C, and 0.5 h for amide reaction at RT). The success of the scaled‐up synthesis was confirmed by the FT‐IR spectra (Figure S3, Supporting Information), affording the product in 94.0% yield. In comparison to previously reported protocols, we remark that our fluorosurfactants can be obtained under a mild condition and atmospheric environment within a relatively short reaction time. These advantages underscore the significant potential of our method for industrial‐scale production.^[^ ^16^, ^18^, ^19^, ^26^ ^]^

### 2.2 Stability and Biocompatibility of W/O_F_ Microdroplets Using the Synthesized Fluorosurfactants

To investigate surface activity of our fluorosurfactants in W/O_F_ droplets, we measured interfacial tension *γ* between deionized water (DI water) and Novec HFE 7500 oil via pendant drop experiments (Figure S4, Supporting Information). The interfacial tension dropped as the concentration of the three fluorosurfactants increased. It was observed that the interfacial tension was reduced from ≈45.43 to ≈29.64, ≈19.90, and ≈16.83 mN m^−1^ when PFPE(H)‐COOCOOC_2_H_5_, PFPE(H)_2_‐ED_900,_ and PFPE(H)_2_‐PED_600_ were applied, respectively. These results highlight their exceptional efficiency in lowering interfacial energy, a key hallmark of high‐performance of fluorosurfactants. We further calculated the critical micelles concentration (CMC) of the three fluorosurfactants, as summarized in Table S2 (Supporting Information).

Water‐soluble fluorophores are ideal for tracking droplet stability, as changes in fluorescence intensity and droplet size can be directly visualized, allowing straightforward monitoring of droplet integrity. To evaluate the droplet‐stabilizing capability of the synthesized fluorosurfactants, we prepared (W/O_F_) microdroplets containing FITC‐CM‐Dextran at a concentration of 3.0 mg·mL^−1^ in Novec HFE 7500 oil, stabilized with different fluorosurfactants at a concentration of 5.0 mm (above their CMC). This was achieved using a flow‐focusing PDMS microfluidic device (Figures S5a and S6a,b, Supporting Information). The generated microdroplets were collected into a home‐made poly (methyl methacrylate) (PMMA) cell and continuously incubated at RT (Figure S6c,d, Supporting Information).

**Figure 2:**
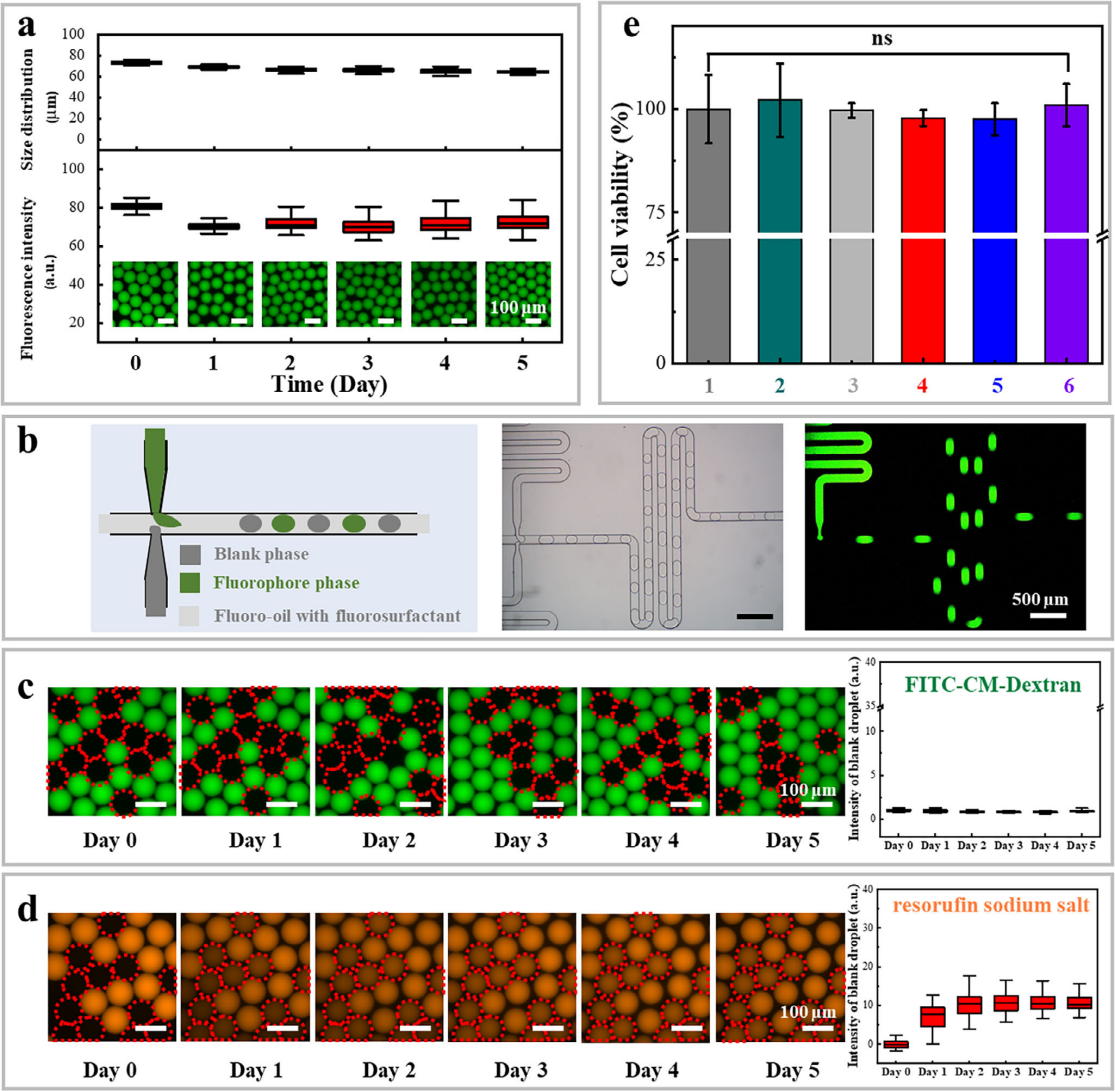
Stability and biocompatibility of microdroplets stabilized by the synthesized fluorosurfactants. a) Size distribution and fluorescence intensity of FITC‐CM‐Dextran in W/O_F_ microdroplets stabilized with PFPE(H)‐COOCOOC_2_H_5_ at the concentration of 5.0 mm over a five‐day stability test at RT. b) Schematic illustration (left), bright‐field micrograph (middle), and fluorescence micrograph (right) of microdroplets with and without fluorophores generated by a double T‐junction generator. c,d) Fluorescence microscopy images, along with a bar chart of fluorescence intensity of blank W/O_F_ microdroplets over five days. Error bars in panels a, c, and d represent 1.5 IQR. Note that the fluorescence intensity was extracted from the raw (gray scale) fluorescence micrographs, with false colors applied to enhance visibility of the distribution and intensity of the fluorophores. For panels a, c, and d, we analyzed over 100 microdroplets to obtain average size plots or intensity of fluorescence. Scale bars correspond to the respective sub‐panels. e) Viability of the surfactants on HepG2 cells. Fluorinated oil containing fluorosurfactants was mixed with the cell culture medium (DEME+10% FBS+1% PIS) in 24‐well after 6 h. Note that samples 1–6 correspond to: 1) control, 2) pure Novec^TM^ HFE 7500, 3) 1.0 wt/v% Pico‐Surf, 4) 5.0 mm PFPE(H)‐COOCOOC_2_H_5_, 5) 5.0 mm PFPE(H)_2_‐ED_900_, and 6) 5.0 mm PFPE(H)_2_‐PEG_600_. Ns (*p* > 0.05) indicates considered statistically no significant (ns) for all HepG2 cells.

The size of the microdroplets stabilized with PFPE(H)‐COOCOOC_2_H_5_ remained largely unchanged after 5‐day incubation (upper panel of **Figure** **2**a), and the corresponding fluorescence intensity of the microdroplets decreased by ≈5.2% during the first day of incubation and then remained nearly unchanged (flotation within ± 1.4%) over the following days (bottom panel of Figure 2a), indicating excellent stability. Similar stability in size of microdroplet and the corresponding fluorescence intensity (flotation within ± 2.9%) was observed for microdroplets stabilized with both PFPE(H)_2_‐ED_900_ and PFPE(H)_2_‐PEG_600_ at RT (Figure S7a,b, Supporting Information). Notably, our fluorosurfactants also preserved the stability of low molecular weight fluorophores such as rhodamine 110 chloride and resorufin sodium salt under ambient conditions (Figure S8a–f, Supporting Information). It is worth noting that there was no phase separation or coalescence of the droplets during storage at RT. Although we observed similar fluorescence encapsulation performance for rhodamine 110 chloride (Figure S9a, Supporting Information) and FITC‐CM‐Dex (Figure S9c, Supporting Information) in microdroplets stabilized with commercial fluorosurfactants (e.g., Pico‐Surf), the fluorescence intensity of resorufin sodium salt decayed in Pico‐Surf stabilized microdroplets within two‐day incubation (Figure S9b, Supporting Information), likely due to leakage or bleach of resorufin sodium salt. This comparison demonstrates the superior droplet‐stabilizing properties of our synthesized fluorosurfactants — PFPE(H)_2_‐ED_900_ and PFPE(H)_2_‐PEG_600_ — by preventing loss of the fluorophores under ambient conditions. Moreover, we observed that microdroplets stabilized by PFPE(H)_2_‐ED_900_ and PFPE(H)_2_‐PEG_600_ retained their integrity and size after multiple thermal treatments up to 95 °C (Figure S10a,b, Supporting Information), highlighting the potential of these fluorosurfactants in biochemical applications, such as polymerase chain reaction (PCR)^[^ ^20^ ^]^ and screening assays.^[^ ^29^ ^]^

To assess the performance of our fluorosurfactants in preventing inter‐droplet mass transfer, we further examined the transfer of fluorophores from fluorosurfactant‐stabilized W/O_F_ microdroplets to blank microdroplets (without fluorophores) (Figure 2b; Figure S5b (Supporting Information) and Methods section for droplet generation details. Remarkably, regardless of the fluorosurfactant applied, almost no diffusion of FITC‐CM‐Dextran was observed from the loaded microdroplets to the blank microdroplets over a five‐day period (Figure 2c; Figure S11a,b, Supporting Information). In contrast, inter‐droplet mass transfer of resorufin sodium salt occurred, with transport from the loaded microdroplets to the blank microdroplets (Figure S12a,b, Supporting Information). Both loaded and blank microdroplets retained their original sizes, with no coalescence detected. This behavior can be attributed to the chemically inert nature of the fluorosurfactants, which effectively mitigated inter‐droplet mass transfer, and the high molecular weight of FITC‐CM‐Dextran.

Beyond their effectiveness in inhibiting inter‐droplet mass transfer for large molecular weight species, our synthesized fluorosurfactants also demonstrate promising biocompatibility, an essential feature for potential applications in biological systems.^[^ ^16^ ^]^ We assessed the biocompatibility of the synthesized fluorosurfactants by culturing HepG2 cells with fluorinated oil containing our synthesized fluorosurfactants in a 24‐well plate. The HepG2 cells interacted with the fluorinated oil owing to the oil is heavier than medium solution. The viability of HepG2 cells was further evaluated after 6 h incubation (Figure 2e), which showcased that these synthesized fluorosurfactants are comparable to the control group and commercial fluorosurfactant (Pico Surf). In addition, yeast cells were encapsulated within microdroplets stabilized by the fluorosurfactants, along with growth medium (YPD broth; see Methods section for details). The encapsulated cells were cultured for 24 h (see Experimental Section for experimental details in Supporting Information). Notably, the yeast cells‐maintained mobility within the W/O_F_ microdroplets stabilized by PFPE(H)‐COOCOOC_2_H_5_, PFPE(H)_2_‐ED_900,_ and PFPE(H)_2_‐PEG_600_ (Figure S13a–c, Supporting Information). We observed no adherence of the yeast cells to the microdroplets interface (Video S1, Supporting Information), and their division and proliferation followed the typical pattern of bud formation and gradual bud growth, as previous reported.^[^ ^30^ ^]^ To further demonstrate the biocompatibility of our synthesized fluorosurfactant, HepG2 cells were cultured in microgels formed by droplet microfluidics using our synthesized fluorosurfactants. These results show that HepG2 cells aggregated into microgels within 3 days, indicated that our synthesized fluorosurfactants are well‐suited for cell‐cluster cultivation (Figure S14a–c, Supporting Information). These findings suggest that our synthesized fluorosurfactants hold considerable potential for applications in 3D cell culturing/cluster cultivation and further development of bioengineering materials fabrication.^[^ ^4^, ^31^ ^]^

### 2.3 Tunable Functionalities of the Synthesized Fluorosurfactants

#### 2.3.1 Selective Enrichment of Fluorophores

**Figure 3:**
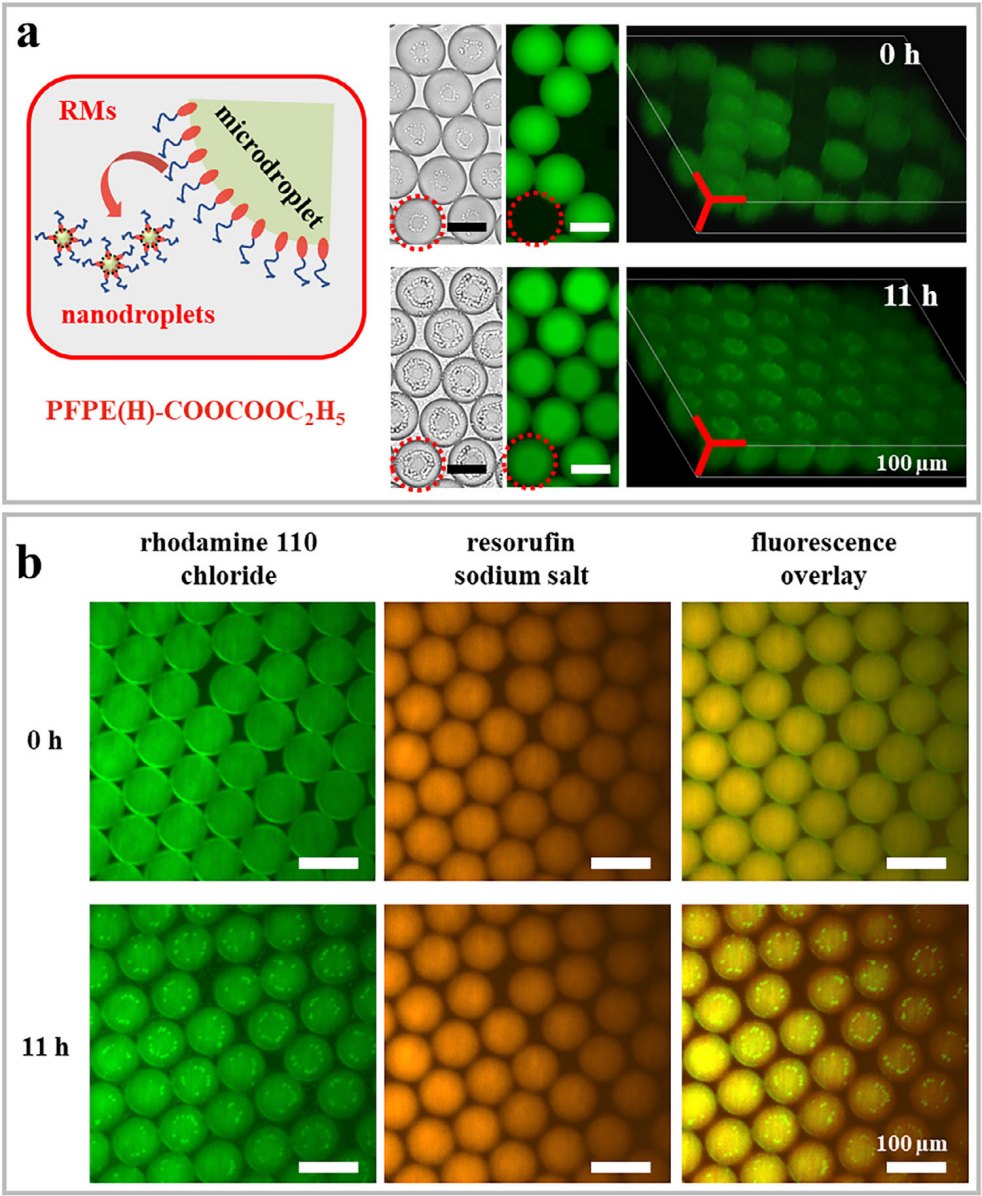
Selective enrichment of fluorophores. a) Schematic illustration of nanodroplets formation via spontaneous emulsification, accompanied by two sets of representative time‐lapse images of microdroplets stabilized by PFPE(H)‐COOCOOC_2_H_5_. Bright‐field (left) and corresponding fluorescence micrographs (middle), along with 3D reconstructions from confocal microscopy images (right), of bi‐microdroplets, both immediately after preparation and after 11 h. Typical microdroplets containing nanodroplets can be clearly visualized as marked in red dashed circles for clarity. b) Time‐lapse fluorescence images showing selective partitioning of rhodamine 110 chloride from PFPE(H)‐COOCOOC_2_H_5_ stabilized parent W/O_F_ microdroplets, containing both rhodamine 110 chloride and resorufin sodium salt, into nanodroplets. Note that fluorescence intensity was extracted from the raw (grayscale) fluorescence micrographs, with false colors applied to enhance the visibility of the distribution and intensity. Scale bars correspond to the respective sub‐panels.

An intriguing observation was the emergence of nanodroplets from parent W/O_F_ microdroplets upon the application of 5.0 mm PFPE(H)‐COOCOOC_2_H_5_. Notably, nanodroplets *did not* form during the initial generation of the parent W/O_F_ microdroplets; instead, they appeared immediately after the parent W/O_F_ microdroplets had formed, regardless of whether fluorophores were encapsulated in them (**Figure** **3**a). In contrast, significantly fewer nanodroplets were observed when PFPE(H)_2_‐ED_900_ or PFPE(H)_2_‐PEG_600_ was applied at the same concentration (Figure S15a,b, Supporting Information).

This observation was further confirmed by time‐lapse experiment, as shown in Videos S2 and S3 (Supporting Information), corresponding to W/O_F_ microdroplets stabilized with PFPE(H)‐COOCOOC_2_H_5_ and PFPE(H)_2_‐ED_900_, respectively. Based on recent studies on nonionic surfactant like Span 80,^[^ ^32^, ^33^ ^]^ we elucidate that hydrogen bonds formed between PFPE(H)‐COOCOOC_2_H_5_ RMs and H_2_O, driven by the high electronegativity of the mixed anhydride in PFPE(H)‐COOCOOC_2_H_5_, facilitated nanodroplets formation through spontaneous emulsification.^[^ ^34^ ^]^ Moreover, we found that rhodamine 110 chloride was selectively partitioned into nanodroplets while resorufin sodium salt remained in the parent W/O_F_ microdroplets (Figure 3b; Figure S16, Supporting Information). We postulate that this selectivity is governed by the hydrophilicity/hydrophobicity of the fluorophores,^[^ ^35^ ^]^ which warrants further investigation in future studies. We remark that such selective enrichment realized by PFPE(H)‐COOCOOC_2_H_5_‐stabilized W/O_F_ microdroplets demonstrates considerable potential for enrichment in microsystems, such as selectively concentrating peptides.^[^ ^36^ ^]^

#### 2.3.2 Engineering Morphology‐Controlled Superstructure Through Nanoparticle Self‐Assembly in Slowly Evaporating Microdroplets

**Figure 4:**
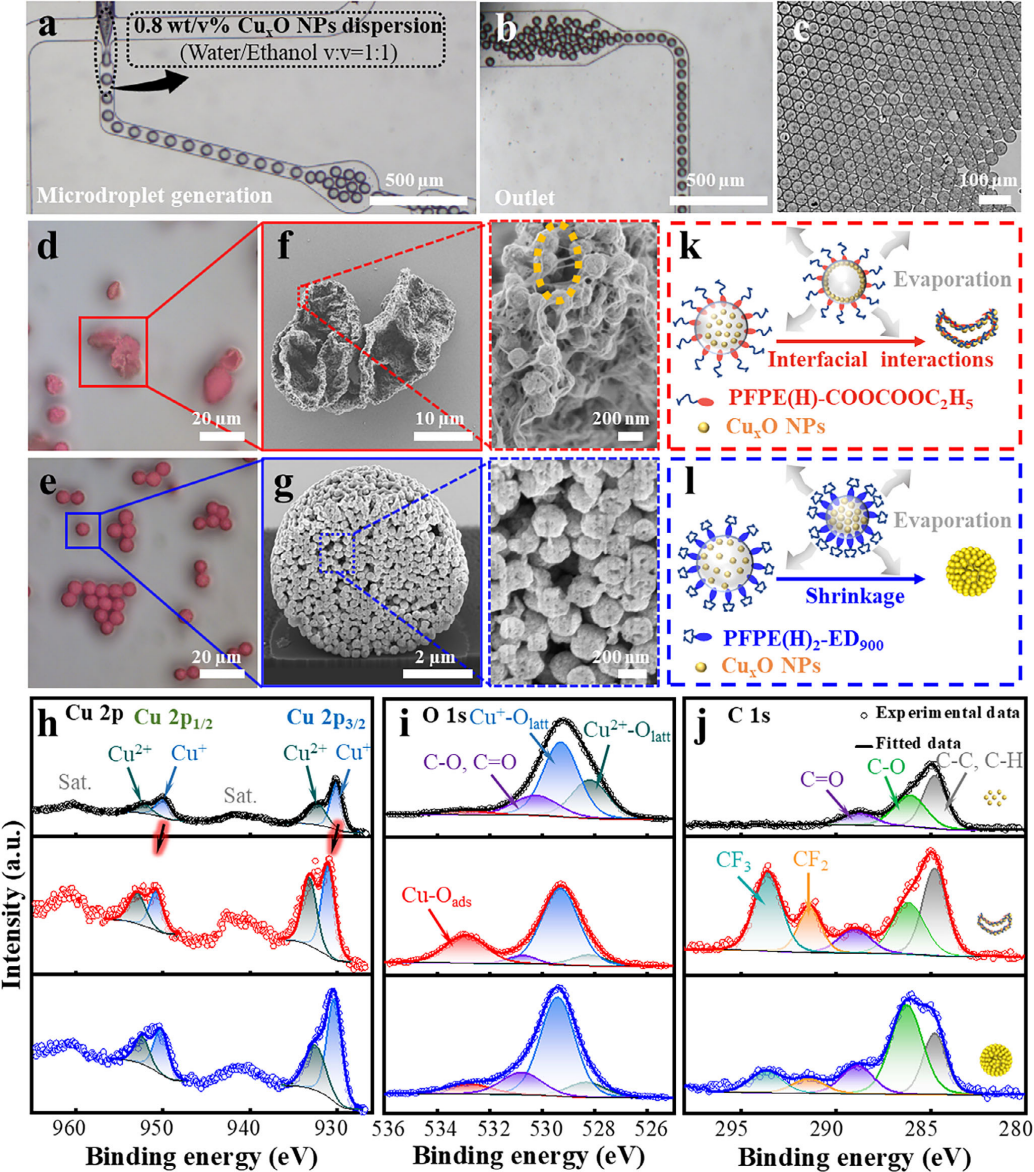
Structure‐controlled self‐assembly of Cu_x_O NPs in slowly drying M/O_F_ microdroplets. a–c) Optical microscopy image of a mixture of water/ethanol in fluorinated oil (M/O_F_) microdroplets formation using a flow‐focus generator (a), outlet of the chip (b), and generated monodisperse M/O_F_ microdroplets (c). d,e) Optical microscopy image of Cu_x_O NP‐colloidosomes (Cu_x_O NP‐CSs) and Cu_x_O NP‐supraparticles (Cu_x_O NP‐SPs). f,g) SEM images of Cu_x_O NP‐CSs and Cu_x_O NP‐SPs. Right panel of f: a zoomed‐in view of the wrinkled surface of the Cu_x_O NP‐CSs, where a yellow ellipse denotes possible presence of PFPE(H)‐COOCOOC_2_H_5_ inside Cu_x_O NP‐CSs. Right panel of g: a zoomed‐in view of the cross‐sectional morphology of a Cu_x_O NP‐SP by focus ion beam (FIB) technique. The Cu 2p h), O 1s i), and C 1s j) XPS spectra of the Cu_x_O NPs (top row of panels h,i,j), Cu_x_O NP‐CSs (middle row of panels h,i,j), and Cu_x_O NP‐SPs (bottom row of panels h,i,j), respectively; k,l) Schematic illustration of possible formation process of Cu_x_O NP‐CSs and Cu_x_O NP‐SPs driven by different fluorosurfactants.

Microdroplets are widely used as confinement systems for colloidal particle self‐assembly, offering an ideal platform for structuring hierarchical materials with diverse applications.^[^ ^37^, ^38^, ^39^, ^40^, ^41^, ^42^, ^43^, ^44^ ^]^ We synthesized polyvinylpyrrolidone (PVP) stabilized copper oxides (Cu_x_O) NPs (Figure S17a,b, Supporting Information), which show great potential in catalysis and optoelectronics.^[^ ^45^, ^46^ ^]^ The synthesized Cu_x_O NPs were then assembled into morphology‐controlled superstructures, facilitated by the slow evaporation of a 1:1 water‐ethanol (*V/V*) mixture in fluorinated oil (M/O_F_) microdroplets stabilized by the fluorosurfactants (**Figure** **4**). In contrast to the coalescence observed in microdroplets stabilized by the commercial fluorosurfactant PFPE(H)‐COOH (Figure S18a,b, Supporting Information), microdroplets containing Cu_x_O NPs remained highly stable with the synthesized fluorosurfactants (Figure 4a‐c). As the inner‐phase solvent evaporated under ambient conditions, the Cu_x_O NPs self‐assembled into hierarchical superstructures, which appeared red in reflective‐mode optical microscopy images (Figure 4d,e).

To understand the role of fluorosurfactants in the formation of the superstructures, we characterized the self‐assembled structures using scanning electron microscopy (SEM). Notably, two distinct superstructures were observed, depending on the fluorosurfactants applied during the self‐assembly process. Specifically, when stabilized with PFPE(H)‐COOCOOC_2_H_5_, the resulting superstructure resembled capsule‐like colloidosomes with wrinkled surface (Cu_x_O NP‐CSs; Figure 4f; Figure S19, Supporting Information). Additionally, we observed a fiber‐like organic matrix, which most likely corresponds to the PFPE(H)‐COOCOOC_2_H_5_ fluorosurfactants, connecting the Cu_x_O NPs (right panel of Figure 4f). In contrast, focused ion beam SEM (FIB‐SEM) imaging revealed that Cu_x_O NPs packed into spherical supraparticles (Cu_x_O NP‐SPs) when PFPE(H)_2_‐ED_900_ was applied (Figure 4g; Figure S20, Supporting Information). Whereas the Cu_x_O NP‐assembly self‐assembled in droplets stabilized by Pico surf exhibited condensed spherical supraparticles (Figure S21, Supporting Information). This structural variation highlights the role of fluorosurfactants in directing the self‐assembly of Cu_x_O NPs from slowly evaporating droplets, as elaborated below.

To investigate the mechanisms driving the formation of two distinct superstructures, we measured the interfacial tension between the fluorinated oil containing different fluorosurfactants and metal‐based colloidal solution, as shown in Figure S22a (Supporting Information). The result demonstrated that the interfacial tension, with different fluorosurfactants, *does not* dominantly govern the construction of different morphologies of Cu_x_O NP‐assemblies. In addition, the zeta potential of Cu_x_O NPs was considered as well. We presented the zeta potential of different colloids in Figure S22b (Supporting Information), including 0.8 wt.% Cu_x_O NPs, 1.0 wt.% SiO_2_ NPs (*d = 200 nm*), 1.0 PS NPs (*d = 280 nm*) dispersed in the mixture solution (water/ethanol = 1:1). All of the zeta potentials of the colloids exhibited negative values. However, the assemblies consisting of SiO_2_ NPs or PS NPs under stabilization of 5.0 mm PFPE(H)‐COOCOOC_2_H_5_ showcased the condensed spherical morphology (shown as the inset images in Figure S22b, Supporting Information), rather than the colloidosome‐like Cu_x_O NP‐assembly. In addition, we analyzed the colloidal self‐assemblies using FT‐IR and X‐ray photoelectron spectroscopy (XPS). FT‐IR spectra of both Cu_x_O NP‐CSs and Cu_x_O NP‐SPs exhibited a prominent C─F bond vibration peak at ≈1254 cm^−1^ (Figure S23a, Supporting Information), confirming fluorosurfactant adsorption on both self‐assemblies.^[^ ^20^ ^]^ Notably, the characteristic peak at 1787 cm^−1^ corresponding to the mixed anhydride C═O stretch of PFPE(H)‐COOCOOC_2_H_5_ disappeared in Cu_x_O NP‐CSs, suggesting a modified C═O bond environment likely caused by the bonding between PFPE(H)‐COOCOOC_2_H_5_ and Cu_x_O NPs. In contrast, the amide C═O stretch at 1695 cm^−1^ of PFPE(H)_2_‐ED_900_ remained almost unaltered in Cu_x_O NP‐SPs, suggesting no significant chemical bonding between Cu_x_O NPs and PFPE(H)_2_‐ED_900_.

The F 1s XPS signal confirms the presence of fluorosurfactants on both Cu_x_O NP‐CSs and Cu_x_O NP‐SPs, aligning well with the FT‐IR measurements (Figure S23b, Supporting Information). The characteristic XPS signal corresponding to Cu^2+^ and Cu^+^ suggests the mixture of Cu_2_O and CuO in both individual NPs and self‐assembled superstructures (Figure 4h). Compared to the individual Cu_x_O NPs, the binding energies of Cu 2p_3/2_ and Cu 2p_1/2_ in Cu_x_O NP‐CSs shifted to higher values by ≈0.9 and 0.73 eV, respectively. In contrast, the shifts in the binding energies of Cu 2p_3/2_ and Cu 2p_1/2_ in Cu_x_O NP‐SPs were less pronounced, with Cu 2p_3/2_ and Cu 2p_1/2_ increasing by ≈0.22 and 0.28 eV, respectively (Figure 4h; Table S3, Supporting Information). This can be ascribed to stronger interaction between the mixed anhydride group of PFPE(H)‐COOCOOC_2_H_5_ with copper oxides compared to that between the amide group of PFPE(H)_2_‐ED_900_ with copper oxides. In the XPS spectra of O_1s_, alongside the signal from lattice oxygen (O_latt_) bonded to the Cu^2+^/ Cu^+^ cations, we observed a peak corresponding to oxygen that is chemically adsorbed (O_ads_) on the surface of the copper oxides NPs. This chemisorbed oxygen exhibits a higher binding energy (Figure 4i).^[^ ^47^ ^]^ We further identified the −CF_3_ and −CF_2_‐ groups in the Cu_x_O NP‐CSs and Cu_x_O NP‐SPs, indicating the presence of the fluorosurfactants (Figure 4j). These results unambiguously proved coordination bonding with different strengths between the fluorosurfactants (i.e., C═O group in the mixed anhydride of the PFPE(H)‐COOCOOC_2_H_5_ or C═O group in the amide bond of the PFPE(H)_2_‐ED_900_ in this study) with copper cations.^[^ ^48^, ^49^ ^]^

We remark that the confined self‐assembly process can be considered an equilibrium, as the drying was slow under ambient conditions. Therefore, kinetic effects should *not* have played a role during the superstructure formation. We propose that the coordination bonding between the PFPE(H)‐COOCOOC_2_H_5_ through the C═O group in the mixed anhydrides and the copper ions, drives accumulation of Cu_x_O NPs at the M/O_F_ microdroplets interface, resulting in voids in the M/O_F_ microdroplets during self‐assembly.^[^ ^50^ ^]^ As a result, capsule‐like Cu_x_O NP‐CSs formed after the evaporation of the inner phase, as illustrated in Figure 4k, which is reminiscence of recent work reported by Fujiwara et al.^[^ ^51^ ^]^ In contrast, for the M/O_F_ microdroplets stabilized by PFPE(H)_2_‐ED_900_, weaker interactions between the Cu_x_O NPs and PFPE(H)_2_‐ED_900_ led to a more homogenous distribution of Cu_x_O NPs inside the droplets, ultimately resulting in the formation of solid Cu_x_O NP‐SPs (Figure 4l). We highlight that the distinct functional groups in our synthesized fluorosurfactants enable versatile modulation of self‐assembled superstructures, thereby offering precise and tunable control over their optoelectronic properties.

## 3 Conclusion

To conclude, we present a facile and robust strategy for synthesizing fluorosurfactants with tunable functionalities through a two‐step reaction. The proposed approach offers distinct advantages compared to the state‐of‐the‐art methods, including less time‐consuming, versatility, less dependent on critical synthetic environments such as high temperature and vacuum. We demonstrated the high stability of droplets enabled by our fluorosurfactants even at elevated temperatures. Additionally, we successfully cultured HepG2 cells and yeast cells in microdroplets, highlighting the bio‐inertness of our synthesized fluorosurfactants. We identified hydrogen bonding between the diblock fluorosurfactant PFPE(H)‐COOCOOC_2_H_5_ and H_2_O as the driving force behind nanodroplets formation, enabling selective transfer of fluorophores in a binary mixture, and opening up a new avenue for enrichment in biosystems. Furthermore, by modulating the interactions between the NPs and fluorosurfactants, we constructed colloidal superstructures with distinct morphologies. The functionalities of our synthesized fluorosurfactant has been summarized in Table S4 (Supporting Information). We envisage that the proposed synthetic route will facilitate the robustness and functionality of fluorosurfactants, and driving advancements in a broad range of droplet‐based applications.

## 4 Experimental Section

### Materials

All chemicals were used as provided without further purification unless noted otherwise. DuPont Krytox 157‐FSH (carboxylated perfluoropolyether, PFPE(H)‐COOH, *M*_w_ = 7000–7500 g mol^−1^) was purchased from Miller‐Stephenson Chemical Co. Inc. (Danbury, CT, USA). 3 m Novec HFE 7100 and 3 m Novec HFE 7500 were purchased from Miller‐ 3 m (St. Paul, MN, USA). Jeffamine ED_900_, triethylamine (TEA, ≥ 99.5%), N‐methylmorpholine (NMM, 99%), benzotrifluoride (BTF), rhodamine 110 chloride, resorufin sodium salt, FITC‐CM‐Dextran (150 KDa), CuSO_4_·5H_2_O (≥ 98.0%), ethylene glycol (≥ 99.0%), YPD Broth, and OptiPrep (D1556) were purchased from Sigma‐Aldrich. PEG‐diamine with a molecular weight of 600 g·mol^−1^ (PEG_600_) was obtained from Shanghai Yare Biotech, Co., Ltd (Shanghai, China). Deionized (DI) water (18.2 MΩ·cm) was prepared using a Milli‐Q Plus water purification system (Milli‐Q Plus water purification, Sichuan Wortel Water Treatment Equipment Co. Ltd, Chengdu, China). All other chemicals were analytical grades and received from Guangzhou Chemical Reagent Co., Ltd. (Guangzhou, China).

### Synthesis of Diblock‐ and Triblock Fluorosurfactants

The fluorosurfactants reported in this work were synthesized using a two‐step process involving mixed anhydride and amidation reactions.^[^ ^52^ ^]^ In a typical synthesis of the diblock fluorosurfactant (PFPE(H)‐COOCOOC_2_H_5_), 157‐FSH (1.0 mmol) and NMM (1.2 mmol) in 3 m Novec HFE 7100 (10.0 mL) were mixed in a round‐bottom flask with stirring, followed by the addition of ethyl chloroformate (1.2 mmol) at 0 °C for 0.5 h. The triblock fluorosurfactants were synthesized by adding the above mixture to a mixture solution containing Jeffamine ED_900_ or PEG_600_‐diamine (0.5 mmol) and triethylamine (1.2 mmol) in BTF (6.0 mL) with stirring at 0 °C for 10 min. The reaction was kept at RT (25 °C) with stirring for 0.5 h. The synthesized diblock‐ and triblock fluorosurfactants were purified by washing with ethanol 5 times, and dried under vacuum at 60 °C for 24 h to yield a transparent, viscous liquid.

### Synthesis of Cu_x_O NPs

166 nm Cu_x_O NPs were synthesized according to previous protocol with minor modification (Figure S15, Supporting Information).^[^ ^53^ ^]^ 0.2496 g of CuSO_4_ and 0.04 g of PVP were dissolved in 50 mL of the ethylene glycol in a 250 mL three‐necked flask under an ultrasonic agitation for 30 min. Next, 25 mL of an aqueous solution containing 0.1 g of NaOH was added to the above solution and stirred for 10 min to ensure complete mixing. A glucose solution (6.0 g in 25 mL of DI water) was then added dropwise to the mixed solutions over 15 min while stirring slowly. The obtained mixture was transferred to a water bath and kept at 80 °C for 2.0 h. Finally, the resulting orange sediment was purified with a centrifugation force of 6 940 × *g*, and washed three times using water and ethanol, respectively. The Cu_x_O NP dispersion was stored in ethanol. XPS results confirmed the presence of both Cu^+^ and Cu^2+^, indicating partially oxidation during sample storage. Consequently, the synthesized NPs were designated as Cu_x_O NPs.

### Polydimethylsiloxane (PDMS) Droplet Generator Fabrication

The PDMS droplet generators with either a flow‐focusing geometry (Figure S4a, Supporting Information) or a double T junction geometry (Figure S4b, Supporting Information) were fabricated using the standard soft lithography technique, as described in the previous article.^[^ ^54^ ^]^ The mold was created from a silicon wafer with the SU‐3050 photoresist to form the designed channel. The based and curing agent was mixed under stirring with a mass ratio of 10:1, followed by degassed in a vacuum chamber until interior bubbles disappeared. Then, PDMS in liquid form was prepared by mixing degassed based and curing agent to duplicate the pattern on the mold. Both PDMS slide and glass cover slide were treated in a plasma cleaner (PDC‐002, Mycro Technologies Co., Ltd, Beijing, China) at a power of 10 W for 60 s, then bonded face‐to‐face to assemble a microfluidic chip. Finally, a hydrophobic microchannel was obtained by injecting the Aquapel (PPG Industries, PA, USA) into the chip, then flushed with air and heated at 120 °C for 0.5 h.

### Stability of W/OF Microdroplets

To test the stability and fluorophores retention at RT, W/O_F_ microdroplets containing fluorophores were generated using a PDMS generator with a flow‐focusing geometry (Figure S5a,b, Supporting Information). Two syringe pumps were used to inject a continuous phase containing Novec HFE 7500 and 5.0 mm fluorosurfactants, and dispersed phase, separately. The flow rate for the continuous phase and disperse phase was set to 5.0 and 1.0 µL min^−1^. The generated microdroplets were then incubated in a home‐made poly (methyl methacrylate) (PMMA) cell for five days (Figure S5c,d, Supporting Information).

To investigate thermal stability of the microdroplets stabilized by the synthesized fluorosurfactants, the microdroplets were subjected to 65 °C for 2.0 h, followed by recovering to RT. The size of the thermal‐treated microdroplets were then measured at RT by optical microscope. Moreover, thermal was applied cycling treatment on the microdroplets to test their stability, which was crucial for various applications such as polymerase chain reaction (PCR).^18^ The thermal cycling program began by ramping the temperature from RT to 65 °C at a rate of 2.0 °C/s, holding at 65 °C for 40 s. The temperature was then raised from 65 to 95 °C, maintained at 95 °C for 15 s, then lowered back to 65 °C at 2.0 °C/s and held for another 15 s. This was followed by 34 cycles oscillating the temperature between 65 and 95 °C. Finally, the system was cooled to RT, after which the microdroplet size was measured.

To further study the inter‐droplet mass transfer, bi‐W/O_F_ microdroplets, containing fluorescent microdroplets and blank microdroplets (without fluorophores), were generated by a PDMS generator with a double T junction geometry. Three syringe pumps were employed to inject two disperse phases and continuous phase, consisting of Novec HFE 7500 with 5.0 mm fluorosurfactants, into the double T‐junction PDMS chip. The flow rate was set to 6.0 µL· min^−1^ for continuous phases, and 0.6 µL· min^−1^ for disperse phase with pure DI H_2_O, and 0.5 µL· min^−1^ for fluorophore disperse phase, respectively. The generated microdroplets were collected for incubation in a home‐made PMMA cell for five days.

### Biocompatibility Test—Viability of HepG2 Cells Assessment

HepG2 cells were cultured in a medium (DEME+10% FBS+1% PIS). Cell viability was assessed using the CCK‐8 assay. Briefly, 500 µL of HepG2 cell suspension (2 × 10^5^ cells· mL^−1^) was seeded into a 24‐well plate and incubated for 1 h to allow cell adhesion to the bottom. Then, remove the medium solution and add the mixture reagent (1 equiv. cck‐8 reagent and 10 equiv. medium) to work on the HepG2 cells for 1.5 h. The culture medium was then removed and replaced with a mixture of CCK‐8 reagent and medium (final ratio: 1 equiv. CCK‐8 to 10 equiv. medium), which was incubated with the cells for 1.5 h. The reacted solution was transferred to a 96‐well plate for absorbance measurement using a UV–vis spectrophotometer under a 450 nm emission wavelength. The remaining HepG2 cells were washed three times with PBS buffer, followed by the addition of 500 µL of fresh medium and 100 µL of fluorinated oil containing the synthesized fluorosurfactants. After incubation for 6 h, the CCK‐8 assay was repeated as described above. Note: All HepG2 cell cultures were maintained in a humidified cell incubator at 37 °C with 5% CO_2_.

### Biocompatibility Test—Yeast Cells Culture

Yeast cells (0.1 g, Angel yeast Co., Ltd., China) were dispersed in YPD broth medium (10 mL, 50 g· L^−1^) at 30 °C for 15 h. The yeast cell suspension was then diluted with YPD broth medium with a concentration of 0.5 mg· mL^−1^. To prepare stable emulsion droplets containing yeast cells, the disperse phase here was yeast cells in YPD broth medium mixed with 15% OptiPrep (D1556). Novec HFE 7500 oil containing synthesized fluorosurfactants at the concentration of 5.0 mm was applied as continuous phase. Note that OptiPrep was a biocompatible and non‐invasive reagent that increases the density of the aqueous phase, thereby preventing cell aggregation.^[^ ^55^ ^]^ The W/O_F_ microdroplets containing yeast cells were generated using a flow‐focus droplet generator. Two syringe pumps were applied to regulate the flow of two phases. The flow rates of continuous and phase and dispersed phase were set at 5.0 and 1.5 µL· min^−1^, respectively. The generated W/O_F_ microdroplets were collected within a PMMA cell, which were subsequently sealed for continuous observation and culturing under ambient conditions.

### Biocompatibility Test—HepG2 Cells Aggregation

The alginate precursor solution containing HepG2 cells (1 × 10^7^ cells mL^−1^), 1.0 wt.% RGD‐alginate, 2 µm cell tracker orange CMRA dye, and 50 mm calcium‐ethylenediaminetetraacetic acid (Ca‐EDTA) was emulsified into microdroplets within fluorinated oil containing the synthesized fluorosurfactant using a flow‐focusing PDMS chip. Two syringe pumps were used to control the continuous and dispersed phases at flow rates 6.0 and 1.0 µL· min^−1^, respectively. The resulting W/O_F_ microdroplets were collected in a fluorinated oil with 0.1 v/v% acetic acid to initiate alginate crosslinking. Following microgel formation, the microgels were transferred into α‐MEM solution supplemented with 25 mm N‐2‐hydroxyethylpiperazine‐N‐2‐ethane sulfonic acid (HEPES) buffer (pH 7.4, kept at 37 °C) to neutralize the acetic acid. The HepG2‐laden microgels were then collected, dispersed in growth medium, and cultured for 3 days. Note: All HepG2‐laden microgels cultures were maintained in a humidified cell incubator at 37 °C with 5% CO_2_.

### Self‐Assembly of Cu_x_O NPs Into Colloidosomes (CSs) and Supraparticles (SPs)

To enable the self‐assembly of Cu_x_O NPs from drying microdroplets, the Cu_x_O NPs were dispersed in a 1:1 water‐ethanol (*V/V*) mixture with a concentration of 0.8 wt/v%. The mixture‐in‐fluorinated oil (M/O_F_) microdroplets were generated using PDMS generator with a flow‐focus geometry, and stabilized by fluorosurfactant at a concentration of 5.0 mm. Two syringe pumps were used to inject the dispersed phase and continuous phase into the flow‐focus PDMS chip, with a flow rate of 5.0 µL· min^−1^ for continuous phases and 2.5 µL· min^−1^ for dispersed phase, respectively. The M/O_F_ microdroplets were subsequently collected in glass vials under ambient conditions, allowing them to dry over ≈5 days at RT. The obtained Cu_x_O NP‐CSs and Cu_x_O NP‐SPs were purified by washing with Novec HFE 7100 oil four times, and were stored in Novec HFE 7100 oil for further characterization.

### Analytic Methods—Fourier Transform Infrared

FT‐IR spectra were recorded on a Vertex 70 FT‐IR spectrometer (Bruker, Germany) in the transmittance mode. The wavenumber range was 400–4000 cm^−1^, and the resolution was 2.0 cm^−1^.

### Analytic Methods—^19^F Nuclear Magnetic Resonance (NMR) Characterization

^19^F NMR spectra were acquired using a mixture of benzene‐D_6_ and hexafluorobenzene with a volume ratio of 1:6 as a solvent via ADVANCE NEO 600 MHz NMR system (Bruker, Germany).

### Analytic Methods—Interfacial Tension Measurements

Interfacial tension γ between water and Novec HFE 7500 oil was measured by Pendant drop technique using an OCA 15 Pro instrument (Dataphysics, Germany) at RT. The water droplet was injected with 0.1 µL· min^−1^ and suspended vertically using a syringe needle into a glass cuvette containing Novec HFE 7500 oil dissolving fluorosurfactants. The measurements were repeated three times for each sample. The interfacial tension γ was calculated by fitting the droplets shape to the Laplace‐Young equation. The resulting data were fit to curves of the form *y*(*x*) = *a*.exp(‐*β*.*x*) + *η*, where the coefficients *a*, *β*, and *η* were extracted using a weighted algorithm implemented in MATLAB.^[^ ^56^ ^]^

### Analytic Methods—Microscopy Image Acquisition and Data Processing

The generation process of W/O_F_ microdroplets was recorded using an inverted optical microscope (Olympus IX2, Tokyo, Japan) equipped with a high‐speed camera (Phantom, MIRO MIIO, Vision Research Inc., Wayne County, NC, USA), operated in bright‐field mode. Fluorescence images were taken by a microscope Leica DMi 5000 m camera with a pE300‐ultra LED illumination system (CoolLED, Andover, UK) using the green LED light (450 nm center wavelength) or the blue LED light (537 nm center wavelength) combined with a BGR filter cube (dichroic mirror: 510 nm). The 3D rendering was reconstructed by Fiji from a series of fluorescence images recorded with a Nikon Eclipse Ti‐U inverted microscope with a Hamamatsu digital camera (ORCA‐flash 4.1, ≈100 fps) and 100 mW 488 and 561 nm excitation laser sources for rhodamine 110 chloride and resorufin sodium salt, respectively. The size distribution of the generated W/O_F_ microdroplets and its fluorescence intensity were analyzed by customized Matlab code.

### Analytic Methods—X‐Ray Photoelectron Spectroscopy (XPS) Measurements

The composition of the synthesized Cu_x_O NPs and Cu_x_O NP‐assemblies were analyzed using X‐ray photoelectron spectroscopy (XPS, ESCALAB 250, USA). In‐depth XPS profiles were obtained by argon‐ion beam etching with a rate of 0.06 nm· s^−1^. All XPS spectra were calibrated to the carbon peak (C 1s, 284.8 eV). The XPS peaks were fitted using CasaXPS software, ensuring that the full widths at the half‐maximum (FWHM) of the main peaks were kept below 2.0 eV, with a fixed Lorentzian/Gaussian ratio of 20%.

### Analytic Methods—Scanning Electron Microscopy (SEM) and Focused‐Ion Beam SEM (FIB‐SEM) Sample Preparation and Measurement

To prepare the sample for SEM, 10.0 µL of Cu_x_O NP‐CSs and Cu_x_O NP‐SPs were drop‐casted on a silicon wafer, and subsequently were dried under ambient condition. The structure of Cu_x_O NPs and Cu_x_O NP‐assemblies were characterized by GeminiSEM 500 (Carl Zeiss Microscopy GmnH) with an accelerating voltage of 0.75 kV in secondary electron (SE) imaging mode. To further analyze the interior morphology of Cu_x_O NP‐SPs, Cu_x_O NP‐SPs was sectioned by focused ion beam (FIB, Thermo Scientific Helios 5 UX) using a Ga ion source. The milled cross‐section was imaged in secondary electron mode at an accelerating voltage of 5.0 kV.

### Analytic Methods—Statistical Analysis

The statistical analysis for the size distribution and fluorescence intensity of the microdroplet was performed by MATLAB R2022B. The viability of HepG2 cells was measured by UV–vis spectrophotometry. p > 0.05 was considered statistically non‐significant (ns) for all HepG2 cells. Interfacial tension and Zeta potential of Colloids was measured three times and calculated the mean ± SD was calculated.

## Conflict of Interest

The authors declare no conflict of interest.

## Author Contributions

L.S. initiated the project. J.Y. designed and synthesized the fluorosurfactants. J.Y., S.H., Y.D., and Z.L. conducted the droplet microfluidics experiments. J.Y., S.X., and Y.D. performed the colloidal self‐assembly experiments. J.Y. analyzed the fluorescence intensity using customized code implemented in MATLAB developed by L.C. All authors contributed to discussion and analysis of the experimental results. J.Y., S.H., S.X., M.J., D.W., S.P., L.S., and L.I. Segerink wrote the manuscript.

## Supporting information



Supporting Information

Supplemental Video 1

Supplemental Video 1

Supplemental Video 1

## Data Availability

The data that support the findings of this study are available from the corresponding author upon reasonable request.
