# Supplementary material for: A Robust Synthesis of Fluorosurfactants with Tunable Functionalities via a Two‐Step Reaction
Source: Adv Sci (Weinh). 2025 Nov 3;12(48):e11811. doi: 10.1002/advs.202511811 (PMC12752586; doi:10.1002/advs.202511811)
Supplement: Supplementary file 1 — Supporting Information [file ADVS-12-e11811-s002.docx]

Supporting Information

**A Robust Synthesis of Fluorosurfactants with Tunable Functionalities via a Two-Step Reaction**

*Jiyuan Yao*^1, 2^, *Shijian Huang*^1^, *Shuting Xie*^1^, *Zhenping Liu*^1^, *Yueming Deng*^1^, *Luca Carnevale*^2^, *Mingliang Jin*^1^, *Loes Irene Segerink*^2^, *Da Wang*^3,^ *, *Lingling Shui*^1^ *, *and Sergii Pud*^2^

^1^ International Joint Laboratory of Optofluidic Technology and System (LOTS), National Center for International Research on Green Optoelectronics, Guangdong Provincial Key Laboratory of Nanophotonic Functional Materials and Devices, South China Academy of Advanced Optoelectronics & School of Optoelectronic Science and Engineering, South China Normal University, Guangzhou, 510006 (P. R. China)

^2^ BIOS Lab-on-a-chip group, EEMCS Faculty, MESA+ institute, University of Twente, Enschede, 7500 AE (The Netherlands)

^3^ South China Academy of Advanced Optoelectronics, South China Normal University, Guangzhou, 510006 (P. R. China)

^*^ Corresponding: [da.wang@m.scnu.edu.cn](mailto:da.wang@m.scnu.edu.cn); [shuill@m.scnu.edu.cn](mailto:shuill@m.scnu.edu.cn)

**This PDF file includes:**

Supporting Figures (Figure S1 to S23)

Supporting Tables (Table S1 to S4)

References (1 to 7)

**Supporting Figures**


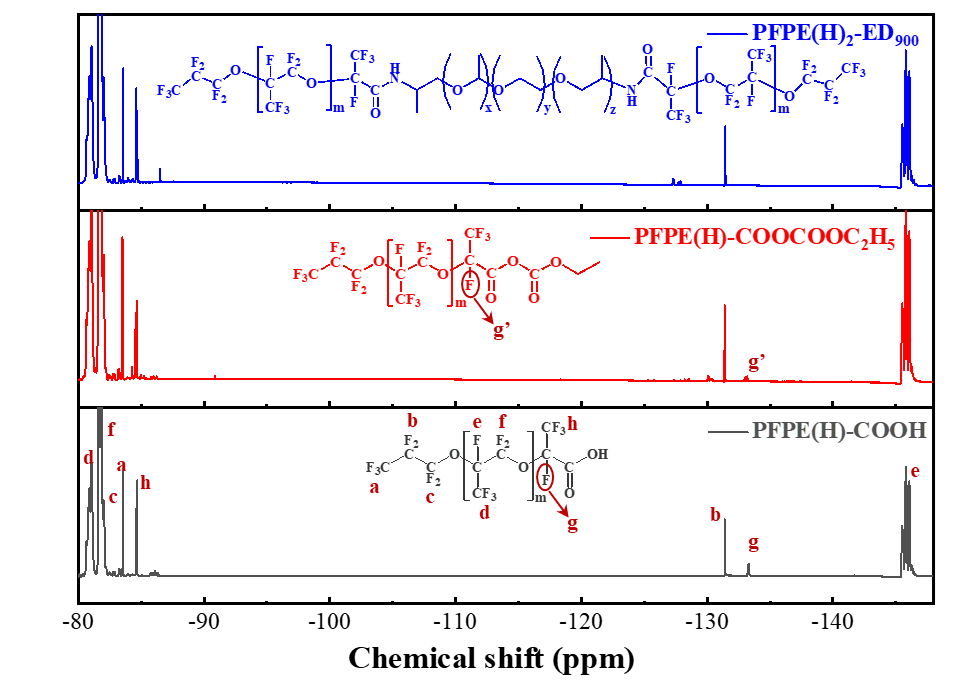


**Figure S1**. Full ^19^F MNR spectra of fluorosurfactants. Full ^19^F MNR spectra of PFPE(H)-COOH (bottom), PFPE(H)-COOCOOC_2_H_5_ (middle), and PFPE(H)_2_-ED_900_ (top), showing distinct chemical shift of fluorine atoms in the fluorosurfactants.


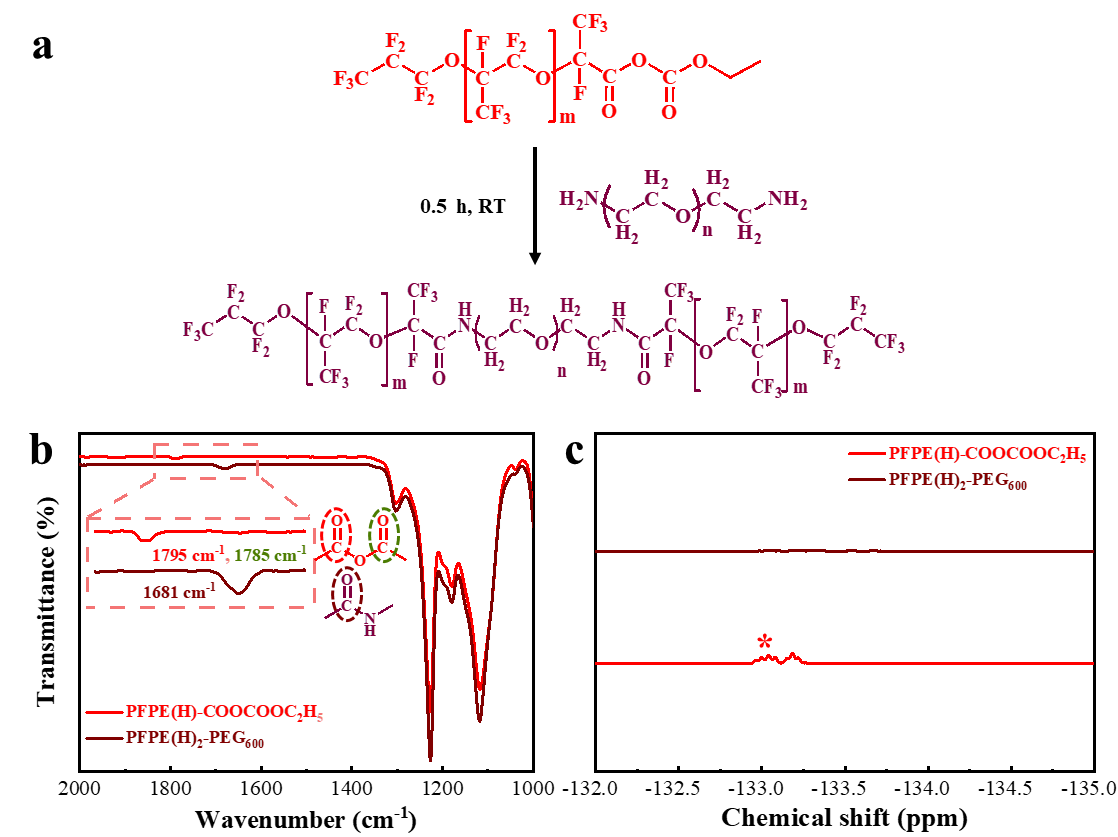


**Figure S2**. Synthesis and characterization of the fluorosurfactant PFPE(H)_2_-PEG_600_. a) Synthetic procedure of the fluorosurfactant PFPE(H)_2_-PEG_600_ *via* amidation reaction. b) FT-IR and c) ^19^F NMR spectra of the diblock fluorosurfactant (PFPE(H)-COOCOOC_2_H_5_) and the triblock fluorosurfactant (PFPE(H)_2_-PEG_600_). Inset of panel b highlights the evolution of vibration peak position of C=O stretch in the fluorosurfactants. Asteroid in panel c denotes fluorine absorption vibration on the adjacent PFPE carboxyl carbon atom in these two fluorosurfactants. This peak disappeared upon the covalently binding of the amino group of Jeffamine ED_900_ (NH_2_-ED_900_-NH_2_) to PFPE(H)-COOCOOC_2_H_5_, confirming the formation of PFPE(H)_2_-PEG_600_.


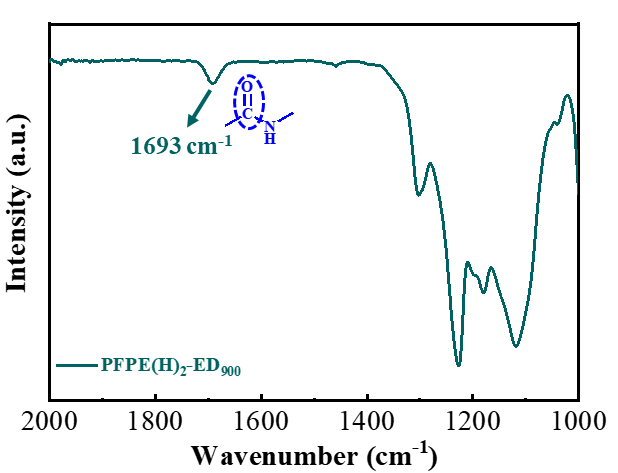


**Figure S3**. FT-IR spectra of the triblock fluorosurfactant (PFPE(H)_2_-ED_900_) in large-scale (25.7 g) synthesis.

**Figure S4.** Interfacial tension (*γ*) measurements for different fluorosurfactants. The data were fitted to lines of the form *y*(*x*) = *α*.*exp*(-*β*.*x*) + *η*, with a weighting given to those data with the smallest variance using a weighted algorithm implemented in MATLAB. Note that error bars represent the standard deviation (n = 3).

**
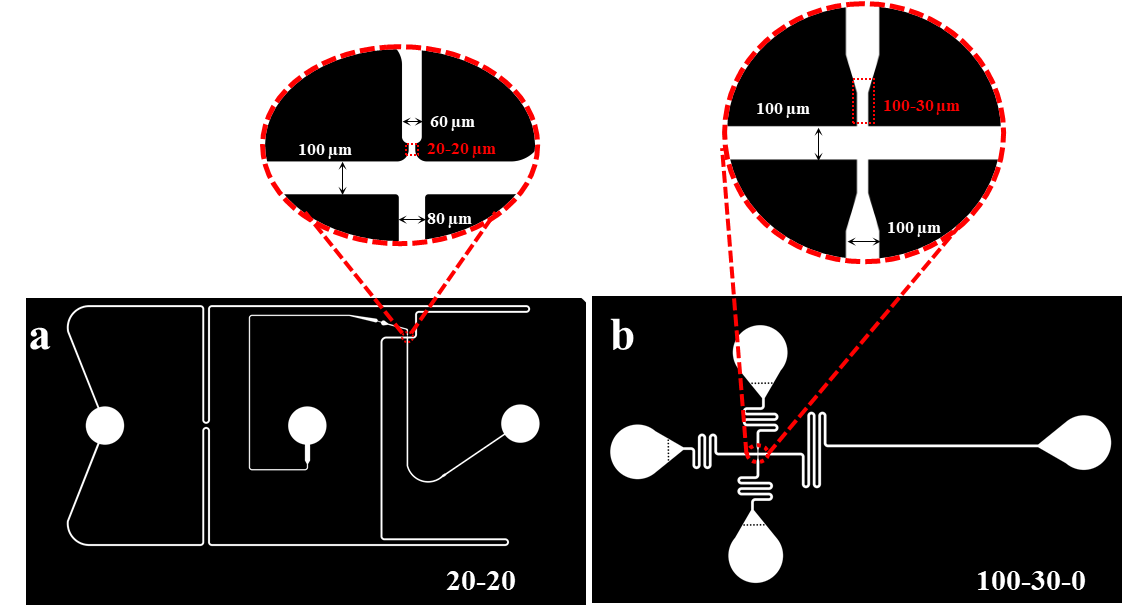
**

**Figure S5.** A CAD file illustrating the design of the microdroplet generator. a) Single W/O_F_ microdroplet generator with a flow-focus junction, with a channel height of 50 µm. b) W/O_F_ bi-microdroplets generator incorporating a double T junction, with a channel height of 50 µm.


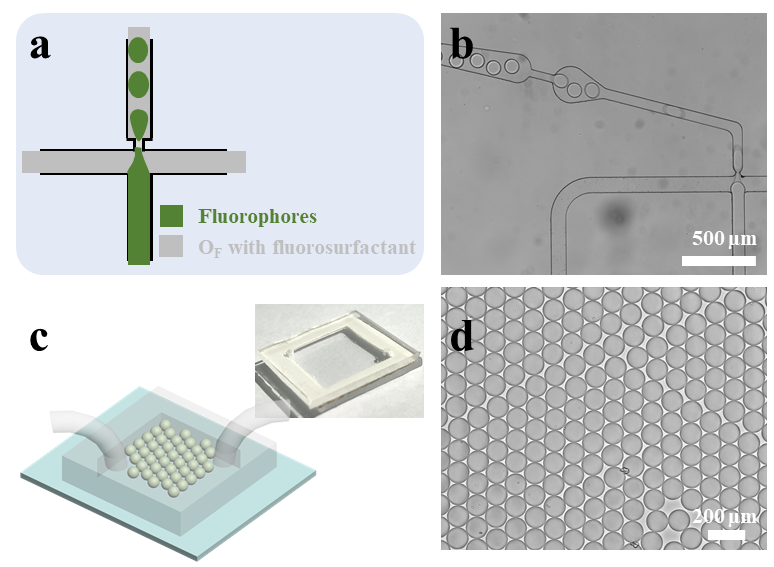


**Figure S6.** Microdroplets generation and incubation. a) Schematic illustration and b) bright-field microscopy image of W/O_F_ microdroplets generation by microchip with a flow-focus junction. c) Schematic illustration and optical micrograph of a PMMA cell for W/O_F_ microdroplet incubation d) Bright-field microscopy image of W/O_F_ microdroplets gathered in a PMMA cell at RT.


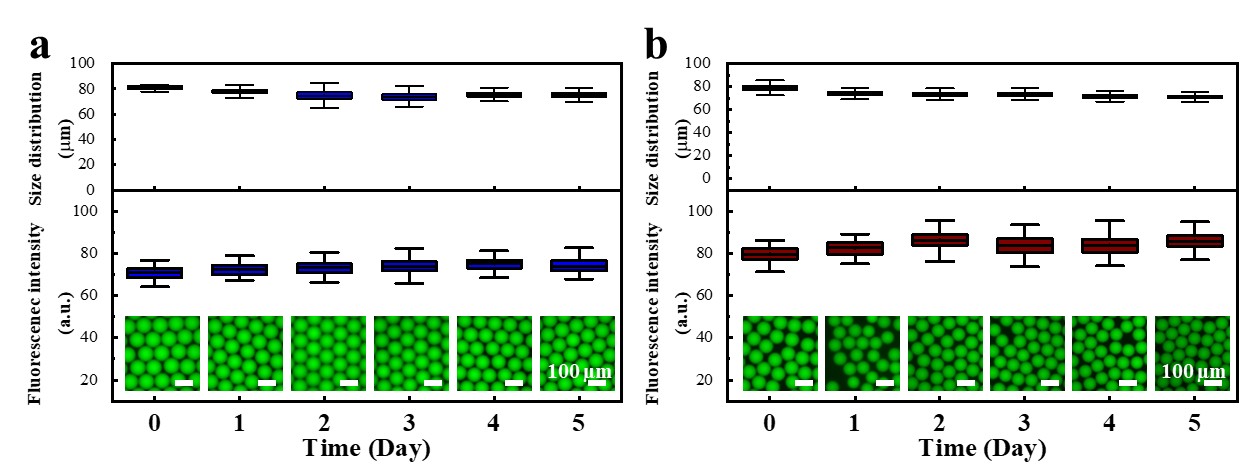


**Figure S7.** Fluorophore retention in W/O_F_ microdroplets. Size distribution and the fluorescence intensity of FITC-CM-Dextran in W/O_F_ microdroplets stabilized with a) PFPE(H)_2_-ED_900_ and b) PFPE(H)_2_-PEG_600_ at the concentration of 5.0 mM over 5 days at RT. Note that the fluorescence intensity was extracted from the raw (gray scale) fluorescence micrographs, with false colors applied to enhance visibility of the distribution and intensity of the fluorophores, and error bars in panel a and b represent 1.5 IQR. For panel a and d, we analyzed over 100 microdroplets to obtain average size plots or intensity of fluorescence. Scale bars correspond to the respective sub-panels.


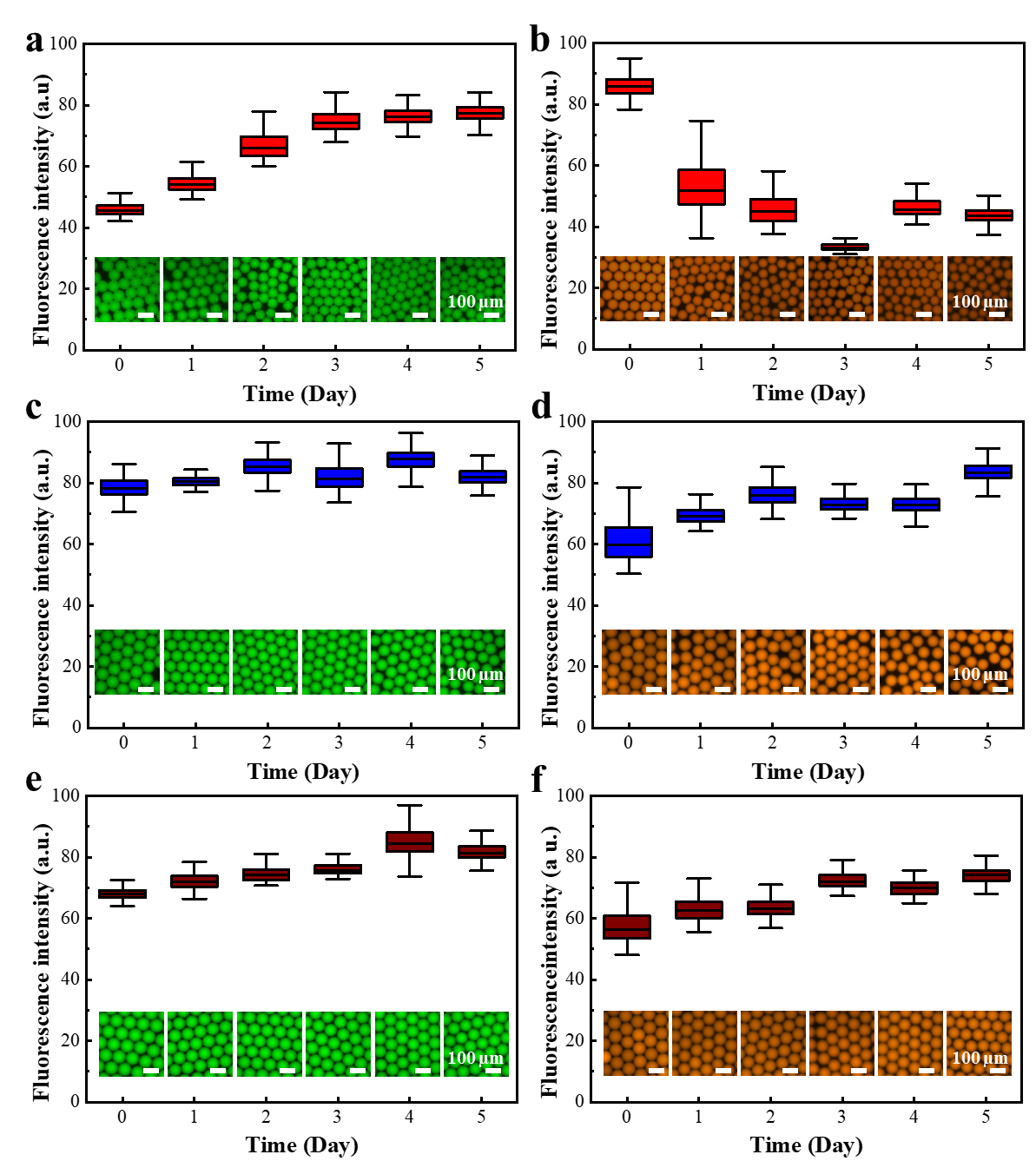


**Figure S8.** Retention of fluorophores with low-molecular weight in W/O_F_ microdroplets. Fluorescence intensity of rhodamine 110 chloride (a, c, e) and resorufin sodium salt (b, d, f) in W/O_F_ microdroplets stabilized by a,b) PFPE(H)-COOCOOC_2_H_5_, c,d) PFPE(H)_2_-ED_900_, e,f) PFPE(H)_2_-PEG_600_ (e-f). Note that the fluorescence intensity was extracted from the raw (gray scale) fluorescence micrographs, with false colors applied to enhance visibility of the distribution and intensity of the fluorophores, and error bars in panel a and b represent 1.5 IQR. For panel a-f, we analyzed over 100 microdroplets to obtain average size plots or intensity of fluorescence. Scale bars correspond to the respective sub-panels.


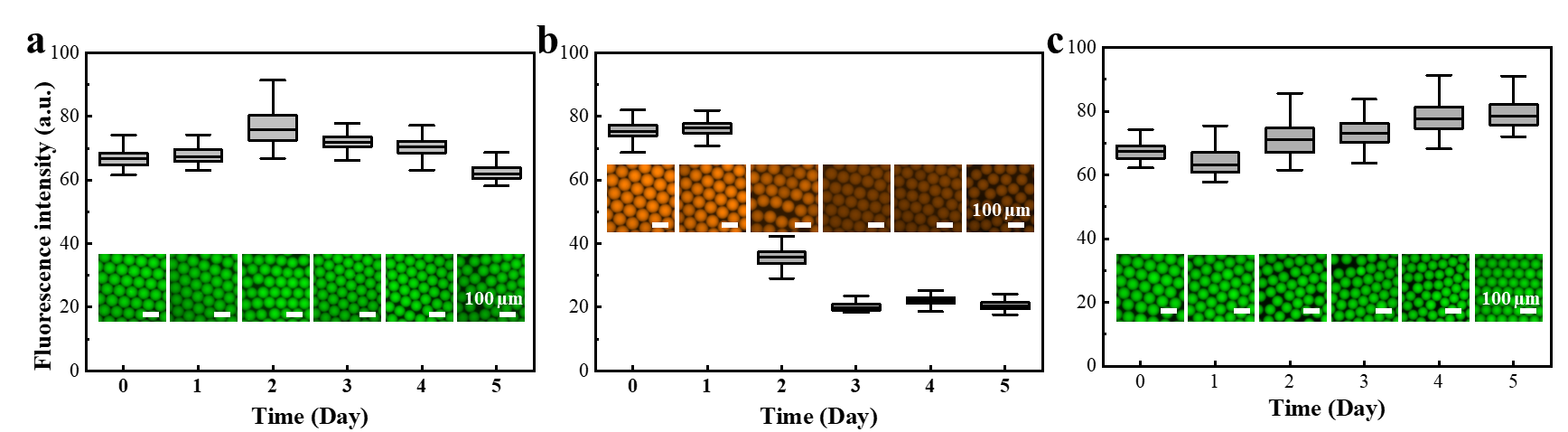


**Figure S9.** Retention of fluorophores in W/O_F_ microdroplets stabilized by commercial fluorosurfactant Pico-Surf™. a) Rhodamine 110 chloride, and b) Resorufin sodium salt, and c) FITC-CM-Dextran. Fluorescence intensity of resorufin sodium salt in W/O_F_ microdroplets stabilized by commercial fluorosurfactant Pico-Surf™ with concentration of 1.0 wt/v% in Novec^TM^ HFE 7500. The fluorescence intensity of resorufin sodium salt drastically decayed when fluorosurfactant Pico-Surf™ was applied. Note that the fluorescence intensity was extracted from the raw (gray scale) fluorescence micrographs, with false colors applied to enhance visibility of the distribution and intensity of the fluorophores, and error bars in panel a and b represent 1.5 IQR. For panel a-f, we analyzed over 100 microdroplets to obtain average size plots or intensity of fluorescence. Scale bars correspond to the respective sub-panels.


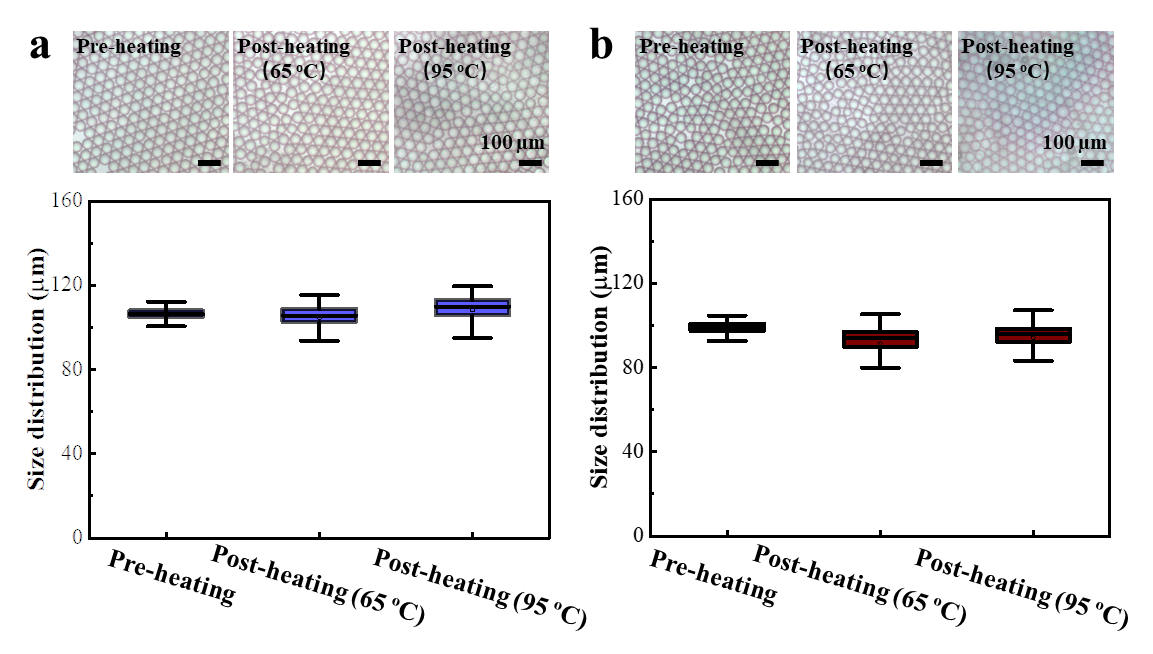


**Figure S10.** Thermal stability of W/O_F_ microdroplets. Bright-field microscopy images and box-plots illustrate size distribution of W/O_F_ microdroplets stabilized by a) PFPE(H)_2_-ED_900_ and b) PFPE(H)_2_-PEG_600_ after isothermal treatment at 65 ºC and thermal cycling between 65 ºC and 95 ºC, demonstrating adequate thermal stability of the microdroplets. Note that size distribution was quantified based on measuring microdroplets after the temperature recovered to RT (25 ºC), and error bars in panel a and b represent 1.5 IQR. For panel and b, we analyzed over 100 microdroplets to obtain average size plots or intensity of fluorescence. Scale bars correspond to the respective sub-panels.

**
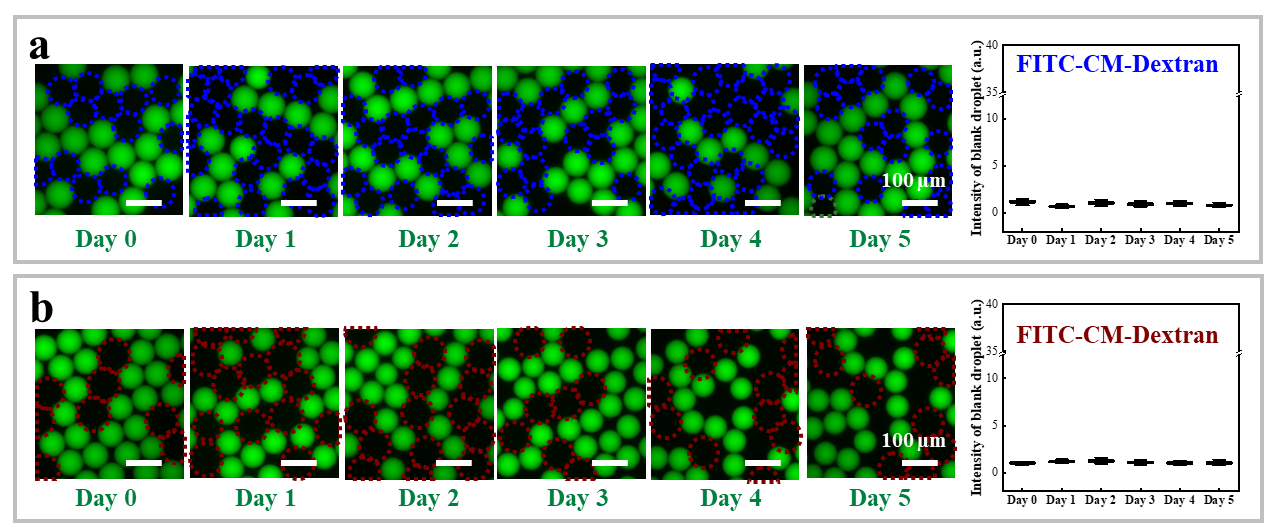
**

**Figure S11.** Inter-droplet diffusion of FITC-CM-Dextran. Bright-field and fluorescence microscopy images of a) PFPE(H)_2_-ED_900,_ and b) PFPE(H)_2_-PEG_600_ stabilized W/O_F_ microdroplets containing FITC-CM-Dextran and pure DI H_2_O (as blank microdroplets) over 5 days. Fluorescence intensity of the microdroplets containing fluorophores and blank microdroplets remained almost identical, indicating that the fluorosurfactants effectively prevented mass transfer at RT. The concentration of PFPE(H)_2_-ED_900_ and b) PFPE(H)_2_-PEG_600_ was 5.0 mM. Note that the fluorescence intensity was extracted from the raw (gray scale) fluorescence micrographs, with false colors applied to enhance visibility of the distribution and intensity of the fluorophores, and error bars in panel a and panel b represent 1.5 IQR. For panel and b, we analyzed over 100 microdroplets to obtain average size plots or intensity of fluorescence. Scale bars correspond to the respective sub-panels.

**
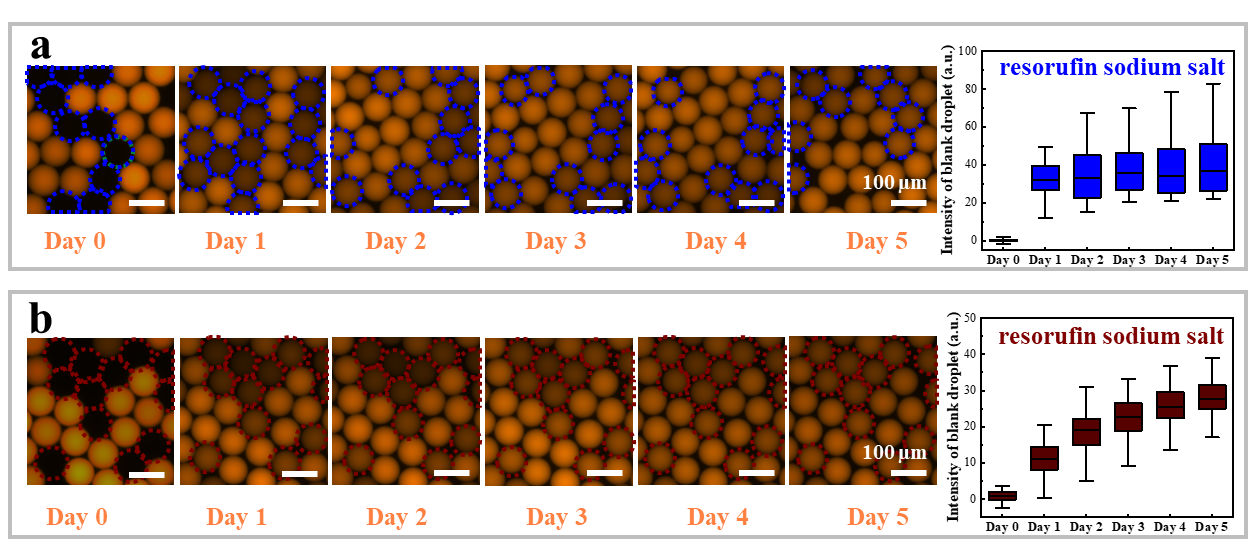
**

**Figure S12. Inter-droplet diffusion of resorufin sodium salt.** a) Bright-field and fluorescence microscopy images of a) PFPE(H)_2_-ED_900,_ and b) PFPE(H)_2_-PEG_600_ stabilized W/O_F_ microdroplets containing resorufin sodium salt and pure DI H_2_O (as blank microdroplets) over 5 days. The concentration of PFPE(H)_2_-ED_900_ and PFPE(H)_2_-PEG_600_ was 5.0 mM. Note that the fluorescence intensity was extracted from the raw (gray scale) fluorescence micrographs, with false colors applied to enhance visibility of the distribution and intensity of the fluorophores, and error bars in panel a and panel b represent 1.5 IQR. For panel and b, we analyzed over 100 microdroplets to obtain average size plots or intensity of fluorescence. Scale bars correspond to the respective sub-panels.


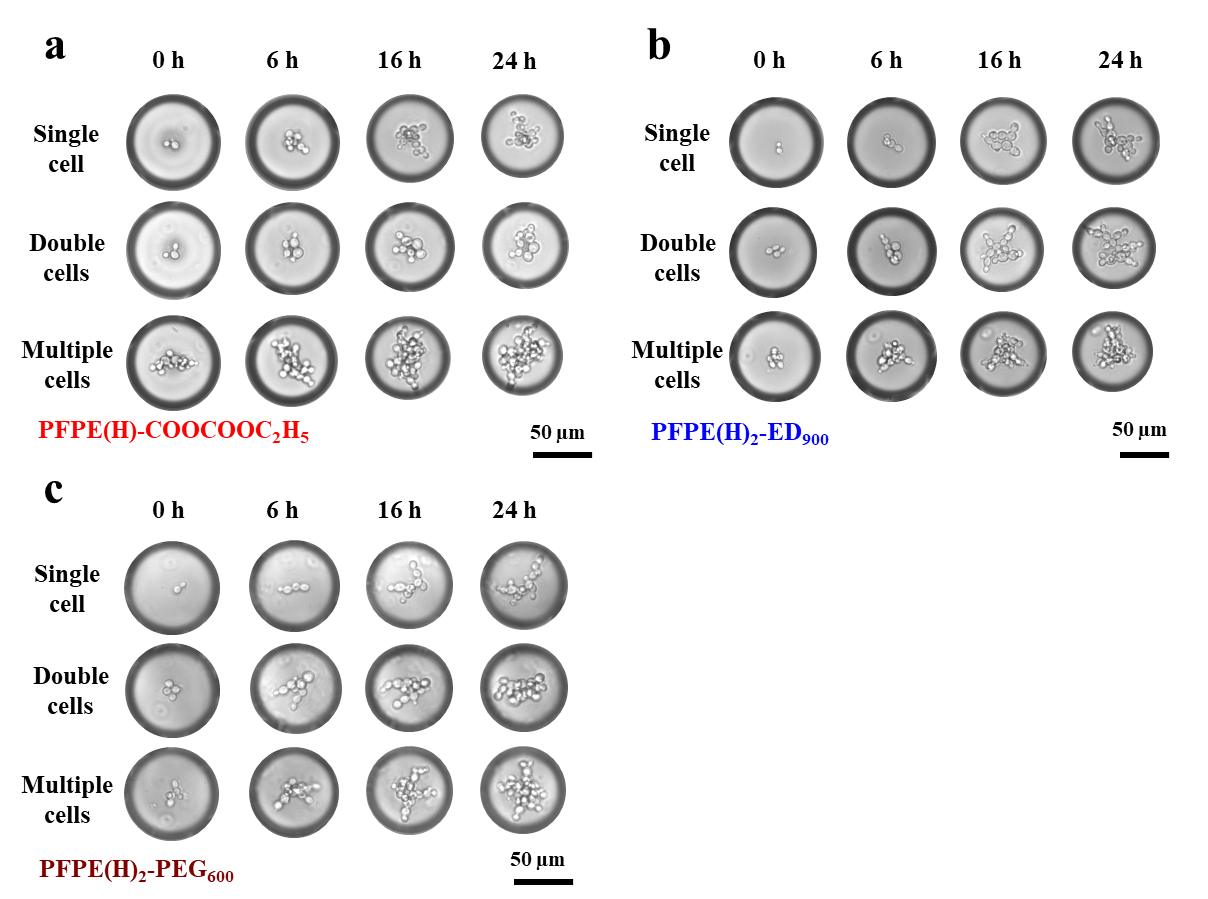


**Figure S13.** Yeast cell culturing in W/O_F_ microdroplets stabilized with the synthesized fluorosurfactants. a) PFPE(H)-COOCOOC_2_H_5_, b) PFPE(H)_2_-ED_900_, and c) PFPE(H)_2_-PEG_600_ at the concentration of 5.0 mM, showing growth of single cell, double cells and multi-cells within individual W/O_F_ microdroplets.


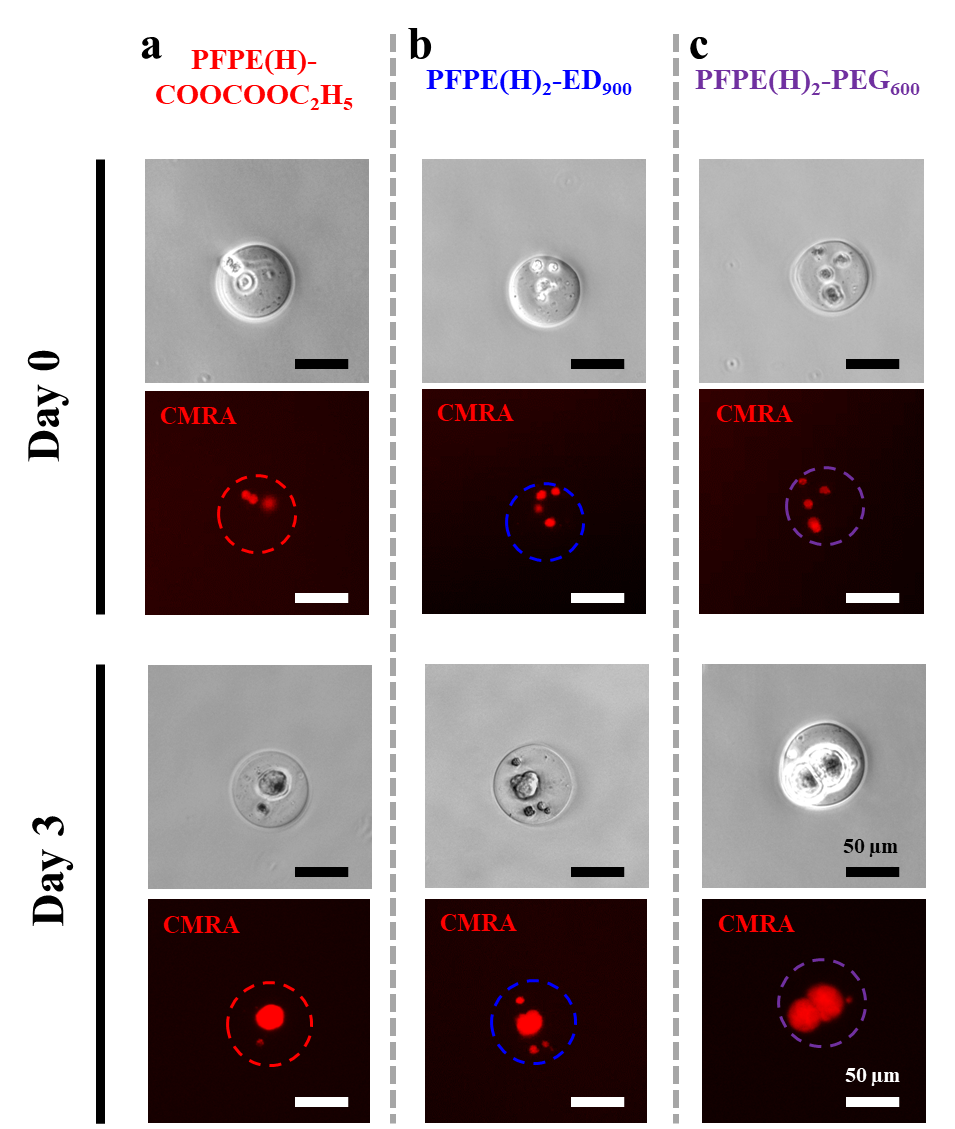


**Figure S14.** HepG2 cell culture and aggregation in hydrogel microspheres fabricated using the synthesized fluorosurfactants. a) PFPE(H)-COOCOOC_2_H_5_, b) PFPE(H)_2_-ED_900_, and c) PFPE(H)_2_-PEG_600_ at the concentration of 5.0 mM. Scale bars correspond to the respective sub-panels.


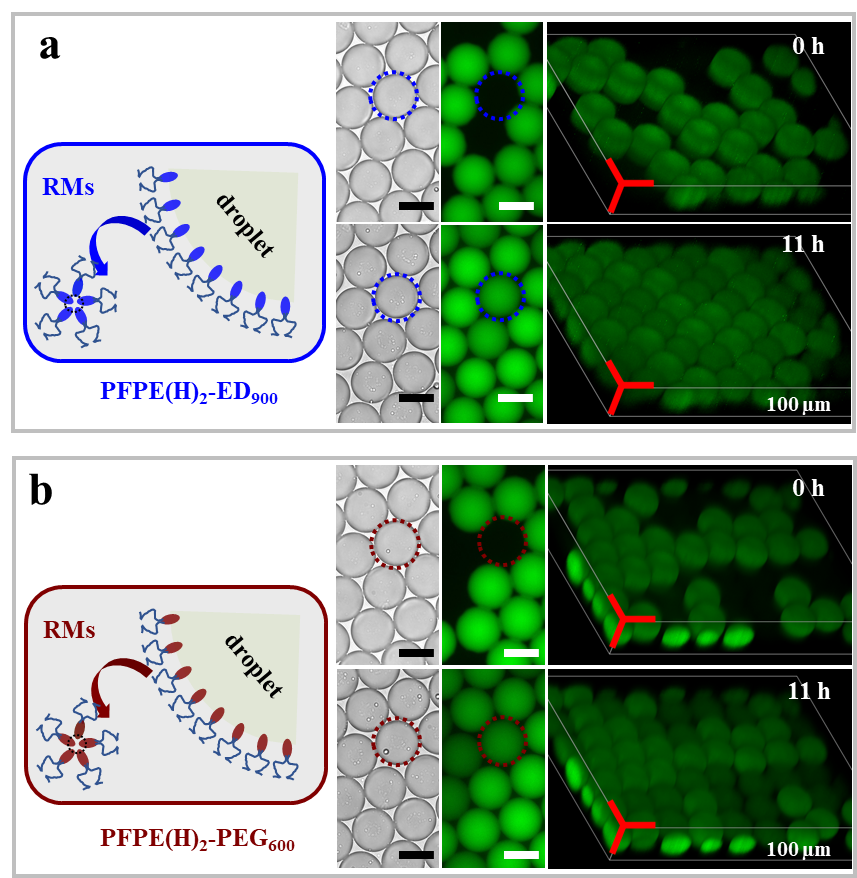


**Figure S15**. Time-lapse measurements of microdroplets stabilized with different fluorosurfactants. The bi-microdroplets containing rhodamine 110 chloride incubated over 11 h, which were stabilized by a) PFPE(H)_2_-ED_900_, and b) PFPE(H)_2_-PEG_600_. Note that the fluorescence intensity was extracted from the raw (gray scale) fluorescence micrographs, with false colors applied to enhance visibility of the distribution and intensity of the fluorophores. In contrast to the results with PFPE(H)-COOCOOC_2_H_5_ was applied, a significant lower amount of nanodroplets were observed. Scale bars correspond to the respective sub-panels.


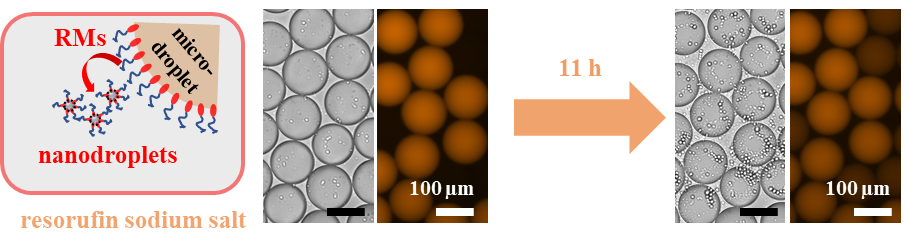


**Figure S16.** Time-lapse measurements of microdroplets containing resorufin sodium salt. Bright-field images and corresponding fluorescence micrographs of microdroplets stabilized with PFPE(H)-COOCOOC_2_H_5_ at concentration of 5.0 mM. Resorufin sodium salt was not observed in the formed nanodroplets. Note that the fluorescence intensity was extracted from the raw (gray scale) fluorescence micrographs, with false colors applied to enhance visibility of the distribution and intensity of the fluorophores. Scale bars correspond to the respective sub-panels.


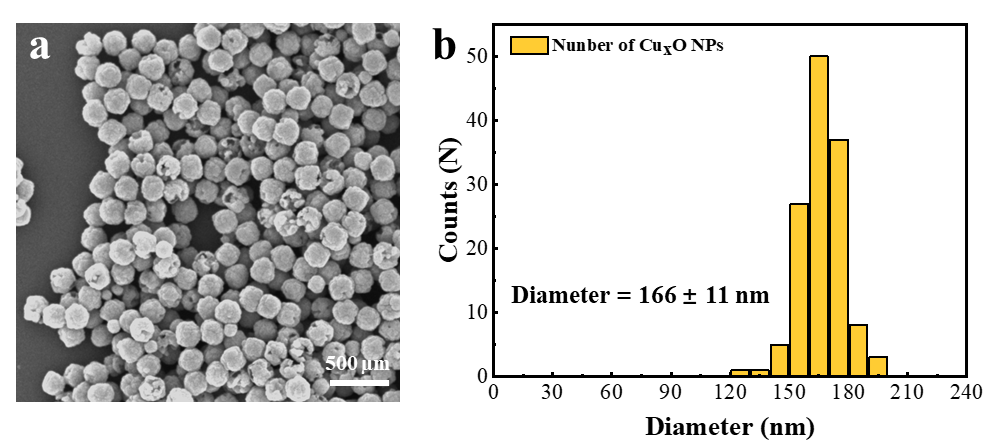


**Figure S17.** Characterization of Cu_x_O NPs. a) Scanning electron microscopy (SEM) image and b) size distribution of the synthesized Cu_x_O NPs.


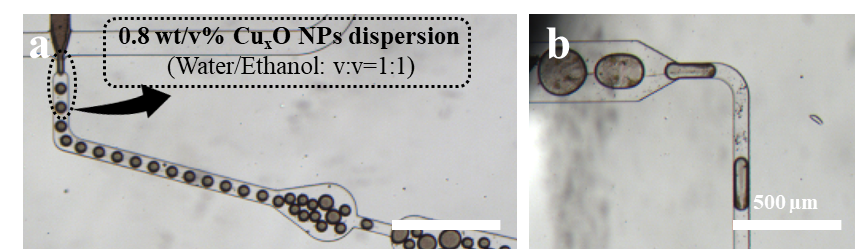


**Figure S18.** Generation of microdroplets containing Cu_x_O NPs using commercial fluorosurfactant. Optical microscopy images for M/O_F_ microdroplets generation in the flow-focus generator with microdroplets (a) and the outlet of the microchip (b). Commercial fluorosurfactant PFPE(H)-COOH at the concentration of 5.0 mM was applied to generate microdroplets, during which droplets coalescence was observed. Scale bars correspond to the respective sub-panels.


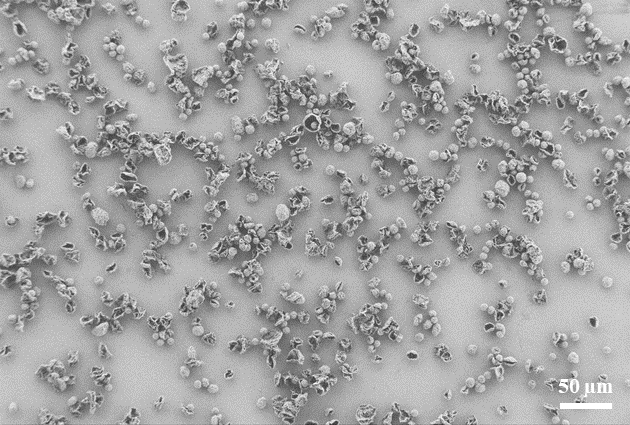


**Figure S19.** Electron microscopy characterization of self-assembled Cu_x_O NP-colloidosomes (CSs). SEM image of Cu_x_O NP-CSs self-assembled from microdroplet stabilized with PFPE(H)-COOCOOC_2_H_5_ at the concentration of 5.0 mM, showing capsule-like morphology.


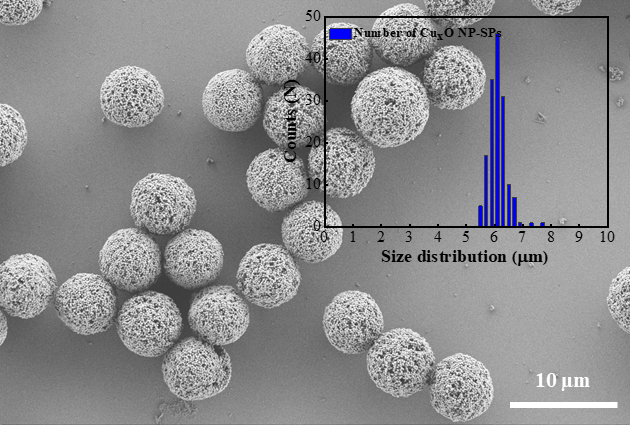


**Figure S20.** Electron microscopy characterization of self-assembled Cu_x_O-NP supraparticles (SPs). SEM image of Cu_x_O NP-SPs self-assembled from microdroplet stabilized with PFPE(H)_2_-ED_900_ at the concentration of 5.0 mM, showing spherical shape. For the size distribution histogram, more than 150 Cu_x_O NPs-SPs were analyzed to obtain the average size distribution.


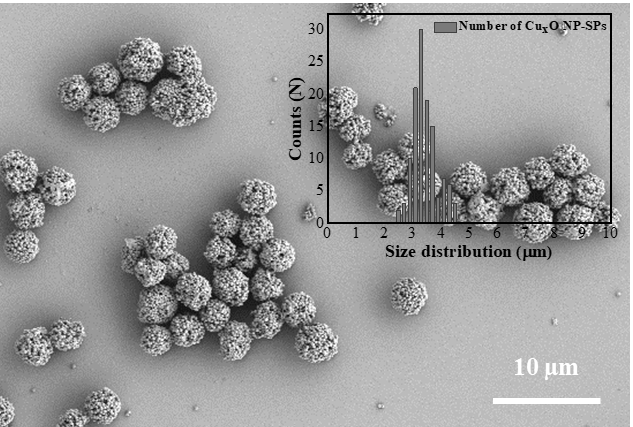


**Figure S21.** Electron microscopy characterization of self-assembled Cu_x_O-NP supraparticles (SPs). SEM image of Cu_x_O NP-SPs self-assembled from microdroplet stabilized with Pico surf^TM^ at the concentration of 2.0 wt%, showing spherical shape. For the size distribution histogram, more than 100 Cu_x_O NPs-SPs were analyzed to obtain the average size distribution.


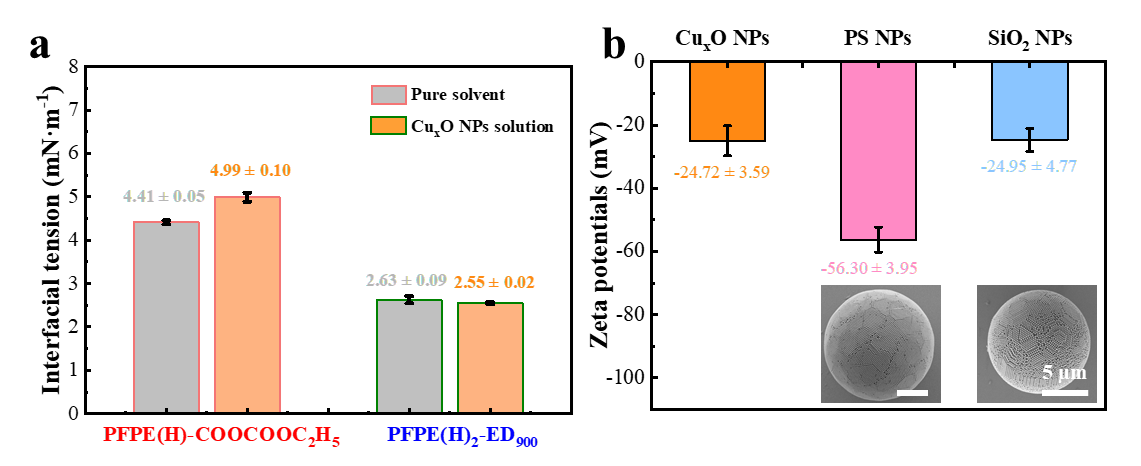


**Figure S22.** Characterization of interfacial tension and zeta potential. a) Interfacial tensions between pure solvents or colloidal suspensions and fluorinated oil containing different fluorosurfactants at a concentration of 5.0 mM. b) Zeta potentials of colloidal suspensions with corresponding SEM images of assemblies. Scale bars in the inset images correspond to the respective sub-panels.


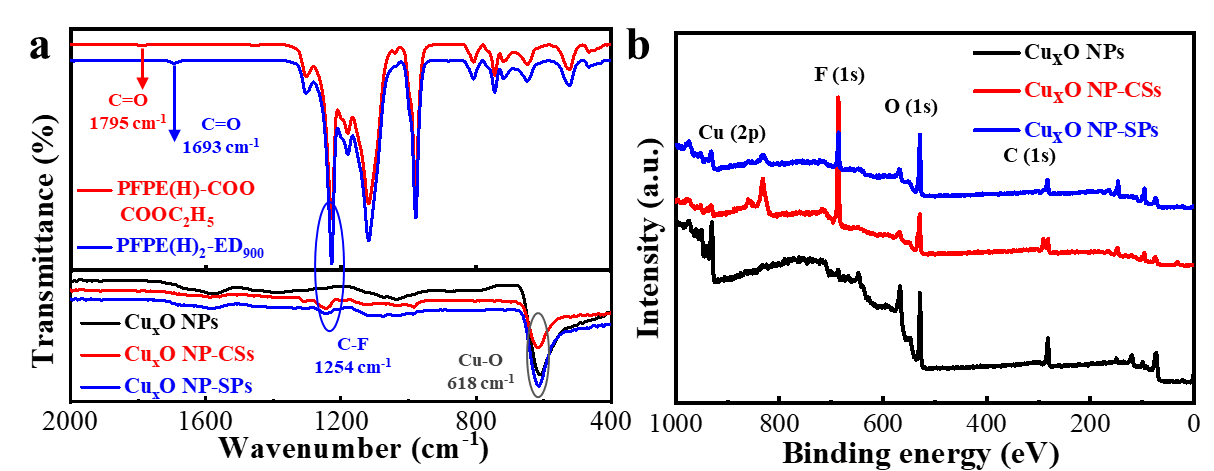


**Figure S23.** FT-IR and XPS characterization of Cu_x_O NPs and self-assemblies. a) FT-IR results demonstrating the presence of the fluorosurfactants on the self-assembled Cu_x_O superstructures. b) Full range XPS spectra of the synthesized Cu_x_O NPs and corresponding superstructures.

**Supporting Tables**

**Table S1.** A direct comparison between literature-reported methods and our strategy.

| **Protocol** | **Time** | **Scalability** | **Specific environment** | **Yields** | **Citations** |
| --- | --- | --- | --- | --- | --- |
| Active *via* **oxalyl chloride** | No details | No details | No details | 80 % | [1] |
|  | 24 h  24 h | No details | Argon gas/50 ^o^C  Argon gas/60 ^o^C | 68%-80 % | [2] |
|  | No details | No details | No details | 80% | [3] |
|  | Overnight  2 days | Different amine-containing compound | Argon gas/RT  Argon gas/50 ^o^C  **OH protection is required** | 75-87% | [4] |
| Controlled/Living Radical Polymerization | 20 h  4 h | Unsaturated compound | RT  Nitrogen gas/70 ^o^C | No details | [5] |
| Electrostatic interaction | Immediately | oppositely charged compound | pH dependent | No details | [6] |
| Active *via* **mixed anhydride** | 0.5 h  0.5 h | Different amine-containing compound | No | 90% | ***This work*** |

**Table S2.** The coefficients *ɑ*, *β* and *η* were listed according to a weighted algorithm in MATLAB, and the concentration and interfacial tension of fluorosurfactants at CMC.

|  | ***ɑ*** | ***β*** | ***η*** | **R^2^** | ***C*_(CMC, mM)_** | **γ_(CMC, mN/m)_** |
| --- | --- | --- | --- | --- | --- | --- |
| **PFPE(H)-COOCOOC_2_H_5_** | 16.92 | 1206 | 26.22 | 0.916 | 2.01 | 29.62 |
| **PFPE(H)_2_-ED_900_** | 22.69 | 4884 | 20.16 | 0.9726 | 1.02 | 19.90 |
| **PFPE(H)_2_-PEG_600_** | 29.04 | 2284 | 14.88 | 0.9761 | 1.23 | 16.83 |

**Table S3.** Summary of binding energies of Cu^+^/Cu^2+^ in Cu_x_O NPs and Cu_x_O NP-superstructures.

|  | **Cu 2p_3/2_**  **(*eV*)** | | **Cu 2p_1/2_**  **(*eV*)** | |
| --- | --- | --- | --- | --- |
|  | **Cu^+^** | **Cu^2+^** | **Cu^+^** | **Cu^2+^** |
| **Cu_x_O NPs** | 930.09 | 932.29 | 949.90 | 952.10 |
| **Cu_x_O NP-CSs** | 931.00 | 933.20 | 950.62 | 952.84 |
| **Cu_x_O NP-SPs** | 930.31 | 932.51 | 950.18 | 952.38 |

**Table S4.** The comparable performance in stability and functionalities of microdroplets between the commercial fluorosurfactant Pico surf^TM^ and our synthesized fluorosurfactants.

| **Fluoro-surfactant** | **Stability** | **Dye encapsulation** | **Satellite droplet formation** | **Cu_x_O NPs adsorption**  **(spheres/colloidosome)** |
| --- | --- | --- | --- | --- |
| PFPE(H)-COOCOOC_2_H_5_ | √ (RT)  × (65 ^o^C) | √ (RT) | √ (RT) | √ (colloidosome) |
| PFPE(H)_2_-ED_900_ | √ (RT)  √ (90 ^o^C) | √ (RT) | × (RT) | × (sphere) |
| PFPE(H)_2_-PEG_600_ | √ (RT)  √ (90 ^o^C) | √ (RT) | × (RT) | × (sphere) |
| Pico surf^TM^ | √ (RT)  √ (90 ^o^C) ^[7]^ | √ (RT) | × (RT) | × (sphere) |

Note: √ indicates assessable; × indicates un-assessable.

**References:**

1. C. Holtze, A. C. Rowat, J. J. Agresti, J. B. Hutchison, F. E. Angilè, C. H. J. Schmitz, S. Köster, H. Duan, K. J. Humphry, R. A. Scanga, J. S. Johnson, D. Pisignanoc, D. A. Weitz, Biocompatible surfactants for water-in-fluorocarbon emulsions, *Lab Chip* **2008**, 8, 1632–1639.
2. Y-L. Chiu, H. F. Chan, K. K. L. Phua, Y. Zhang, S. Juul, B. R. Knudsen, Y-P Ho,K. W. Leong, Synthesis of fluorosurfactants for emulsion-based biological applications, *ACS Nano* **2014**, 8, 3913–3920.
3. O. Wagner, J. Thiele, M. Weinhart, L. Mazutis, D. A. Weitz, W. T. S. Huck, R. Haag, Biocompatible fluorinated polyglycerols for droplet microfluidics as an alternative to PEGbased copolymer surfactants, *Lab Chip* **2016**, 16, 65–69.
4. M. S. Chowdhury, W. Zheng, S. Kumari, J. Heyman, X. Zhang, P. Dey, D. A. Weitz , R. Haag, Dendronized fluorosurfactant for highly stable water-in-fluorinated oil emulsions with minimal inter-droplet transfer of small molecules, *Nat. Commun.* **2019**, 10, 4546.
5. X. Li, S-Y. Tang, Y. Zhang, J. Zhu, H. Forgham, C-X. Zhao, C. Zhang, T. P. Davis, R. Qiao, Tailored fluorosurfactants through controlled/living radical polymerization for highly stable microfluidic droplet generation, *Angew. Chem. Int. Ed.* **2023**, 63, e202315552.
6. C. J. DeJournette, J. Kim, H. Medlenm X. Li, L. J. Vincentm C. J. Easley, Creating biocompatible oil−water interfaces without synthesis: Direct interactions between primary amines and carboxylated perfluorocarbon surfactants, *Anal. Chem.* **2013**, 85, 10556–10564.
7. S. W. Wingett, S. Andrews, P. Fraser, J Morf, RNA proximity sequencing data and analysis pipeline from a human neuroblastoma nuclear transcriptome, *Sci. Data* **2020**, *7*(1), 35.
